# Oncostatin M promotes excitotoxicity by inhibiting glutamate uptake in astrocytes: implications in HIV-associated neurotoxicity

**DOI:** 10.1186/s12974-016-0613-8

**Published:** 2016-06-10

**Authors:** Shamsudheen Moidunny, Marco Matos, Evelyn Wesseling, Santanu Banerjee, David J. Volsky, Rodrigo A. Cunha, Paula Agostinho, Hendrikus W. Boddeke, Sabita Roy

**Affiliations:** Department of Surgery, Division of Basic and Translational Research, University of Minnesota, Minneapolis, MN USA; Center for Neuroscience of Coimbra, Faculty of Medicine, University of Coimbra, Coimbra, Portugal; Department of Neuroscience, University Medical Center Groningen, University of Groningen, Groningen, The Netherlands; Molecular Virology Division, St. Luke’s-Roosevelt Hospital Center, New York, USA; Department of Pharmacology, University of Minnesota, Minneapolis, MN USA

**Keywords:** Glutamate, GLAST, GLT-1, Astrocytes, Oncostatin M, Interleukin 6, NMDA, Excitotoxicity, HIV

## Abstract

**Background:**

Elevated levels of oncostatin M (OSM), an interleukin-6 cytokine family member, have been observed in HIV-1-associated neurocognitive disorders (HAND) and Alzheimer’s disease. However, the function of OSM in these disease conditions is unclear. Since deficient glutamate uptake by astrocytes is instrumental in HAND-associated neurotoxicity, we hypothesized that OSM impairs glutamate uptake in astrocytes and thereby promotes neuronal excitotoxicity.

**Methods:**

Primary cultures of mouse cortical astrocytes, neurons, microglia, and BV2 cell line were used. The expression of glutamate transporters (GLAST/EAAT1 and GLT-1/EAAT2) was investigated using real-time PCR and Western blot, and their activity was assessed by measuring ^3^H-d-aspartate uptake. Neuronal toxicity was measured using the colorimetric MTT (3-(4,5-dimethylthiazol-2-yl-) 2,5-diphenyltetrazolium bromide) assay and immunocytochemistry. A chimeric HIV-1 that infects murine cells (EcoHIV/NL4-3-GFP virus (EcoHIV)) was used to investigate whether the virus induces OSM, OSM receptor (OSMR)-β, glycoprotein 130 (gp130), GLT-1, GLAST (mRNA and protein), and OSM release (ELISA) in cultured BV2 cells, primary microglia, or astrocytes. Statistical analyses of the data were performed using one-way ANOVA (to allow multiple comparisons) and two-tailed Student’s *t* test.

**Results:**

OSM treatment (10 ng/mL) time-dependently reduced GLAST and GLT-1 expression and inhibited ^3^H-d-aspartate uptake in cultured astrocytes in a concentration-dependent manner, an effect prevented by the Janus kinase (JAK)/signal transducers and activators of transcription (STAT)3 inhibitor AG490. Down-regulation of astrocytic glutamate transport by OSM resulted in NMDA receptor-dependent excitotoxicity in cortical neurons. Infection with EcoHIV induced OSM gene expression and protein release in BV2 cells and microglia, but not in astrocytes. Conversely, EcoHIV caused a fivefold increase in OSMR-β mRNA (but not gp130) and protein in astrocytes, but not in microglia, which did not express OSMR-β protein. Finally, astrocytic expression of GLAST gene was unaffected by EcoHIV, whereas GLT-1 mRNA was increased by twofold.

**Conclusions:**

We provide first evidence that activation of JAK/STAT3 signaling by OSM inhibits glutamate uptake in astrocytes, which results in neuronal excitotoxicity. Our findings with EcoHIV suggest that targeting OSMR-β signaling in astrocytes might alleviate HIV-1-associated excitotoxicity.

**Electronic supplementary material:**

The online version of this article (doi:10.1186/s12974-016-0613-8) contains supplementary material, which is available to authorized users.

## Background

Astrocytes are the most abundant cell type of the brain [1], where one of their major functions is to support neurons. They ensure optimal conditions by maintaining local ion and pH homeostasis, regulating neurotransmitters, and clearing metabolic waste [2]. In addition, they store glycogen and supply nutrients to neurons [3]. Dysfunction of astrocytes could therefore have serious consequences for neuron survival. One major cause of neuronal death in the central nervous system (CNS) is excessive release of glutamate and subsequent activation of neuronal *N*-methyl d-aspartate (NMDA) receptors [4, 5]. Astrocytes prevent this neuronal excitotoxicity by sequestration of extracellular glutamate, which is mediated mainly by two Na^+^-dependent transporters: glutamate aspartate transporter (GLAST/EAAT1) and glutamate transporter-1 (GLT-1/EAAT2) [6, 7]. Consistently, loss of astrocytic glutamate uptake and metabolism is one of the key factors responsible for neurotoxicity associated with amyotrophic lateral sclerosis (ALS) [8, 9], Alzheimer’s disease (AD) [10], and HIV-associated neurocognitive disorders (HAND) [11, 12]. In order to develop therapeutic interventions against excitotoxicity associated with these CNS disorders, it is critical to identify factors that regulate astrocytic glutamate transporter expression and activity.

Inflammation of the CNS generally causes a reduction of glutamate transporter expression and consequently dampens glutamate uptake capacity of astrocytes [13, 14]. Thus, pro-inflammatory mediators such as tumor necrosis factor (TNF)-α and interleukin (IL)-1β differentially regulate glutamate transporter expression and glutamate uptake capacity in astrocytes [13–16]; however, the molecular mechanisms that link inflammatory processes and decreased astrocytic glutamate uptake are not well understood. Recent evidence suggest that oncostatin M (OSM), a pleiotropic cytokine belonging to the IL-6 family, might influence neurodegenerative processes associated with a variety of brain diseases. OSM levels have been found to be elevated in both serum and brain lesions of multiple sclerosis (MS) patients [17, 18]. In addition, high levels of OSM are spontaneously secreted by peripheral blood mononuclear cells (PBMCs) isolated from MS, AD, and HAND patients [17, 19, 20]. Although, the precise function of OSM in these disease conditions is unclear, there is limited evidence suggesting that OSM disrupts blood-brain barrier function [21, 22] and mediates pro- and anti-inflammatory effects in the CNS [23–25]. In addition, we have previously shown, along with others, that activation of neuronal OSM receptors protects them against glutamate- and NMDA-induced excitotoxicity [26, 27], potentially by stimulating expression of the neuromodulatory adenosine A_1_ receptors [26]. Furthermore, a recent study showed that overexpression of OSM receptor (OSMR)-β in neurons is protective against ischemic stroke, whereas decreased neuronal OSMR-β expression results in worse stroke outcomes [28]. In contrast, earlier studies demonstrated that OSM actually mediates HIV-associated neurotoxicity in vitro [29]; however, the potential mechanism(s) are not yet understood.

Like neurons, astrocytes may also serve as an important target for OSM in the CNS as its receptor subunits (OSMR-β and glycoprotein 130 (gp130)) are abundantly expressed in these cells [30, 31]. Activation of OSM receptors in astrocytes has been shown to induce reactive astrogliosis [32, 33] and stimulate expression of different matrix metalloproteinases [34] and pro-inflammatory factors including IL-6 [35], prostaglandin (PG) E2, and cyclooxygenase-2 [30]. In addition, OSM has been shown to induce expression of α1-antichymotrypsin (ACT) in astrocytes [36]. ACT is an acute phase protein that has been associated with the formation of amyloid-β deposits found in brain tissue of AD patients [37, 38]. Taken together, these findings suggest that OSM might directly regulate the inflammatory activity of astrocytes in the CNS.

Despite the clear role of OSM in neurocognitive disease, the involvement of OSM in the regulation of glutamate uptake in astrocytes has not yet been addressed. We hypothesized that OSM down-regulates glutamate uptake process in astrocytes and thereby promote neuronal excitotoxicity. Since OSM may play an important role in HIV-1-associated neuropathogenesis [20, 29], we further investigated whether infection with a chimeric HIV-1 (EcoHIV/NL4-3-GFP virus (EcoHIV)) [39, 40] induces the expression of OSM and/or its receptors (OSMR-β and gp130) in cultured microglia and astrocytes.

## Methods

### Chemicals and reagents

^3^H-d-aspartate (specific activity = 10–25 Ci/mmol) was obtained from PerkinElmer (MA, USA) and Aquasafe 500 Plus liquid scintillation cocktail from Zinsser Analytic (Frankfurt, Germany). Neurobasal media, Hank’s buffered salt solution (HBSS), phosphate-buffered saline (PBS), sodium pyruvate, l-glutamine, penicillin-streptomycin, 4-(2-hydroxyethyl)-1-piperazineethanesulfonic acid (HEPES), glutaMAX-1, and B27 supplement were from Gibco (Breda, The Netherlands). Dulbecco’s modified Eagle medium (DMEM) media and fetal calf serum (FCS) were from PAA Laboratories (Cölbe, Germany). Trypsin was obtained from Life Technologies (Breda, The Netherlands). All other cell medium components, recombinant mouse OSM, recombinant mouse IL-6, d-aspartate, l-leucine methyl ester (LME), *N*-methyl-d-glucamine (NMG), and the dyes used to stain cell nuclei (Hoechst 33342 and propidium iodide) were purchased from Sigma-Aldrich (Zwijndrecht, The Netherlands). DL-*threo*-β-benzyloxyaspartic acid (TBOA) and MEK1/2 inhibitor U0126 were obtained from Tocris Bioscience (Bristol, UK). Janus kinase (JAK) inhibitor (AG490) and phosphatidylinositol 3-kinase (PI3K) inhibitor (LY294002) were obtained from Calbiochem (CA, USA). Reagents used in immunoblotting experiments were purchased from Bio-Rad Laboratories, except polyvinylidene fluoride (PVDF) membranes that were obtained from Millipore (Amsterdam, The Netherlands). Primary antibodies, mouse monoclonal anti-β-actin, rabbit polyclonal anti-GLT-1/EAAT2, rabbit polyclonal anti-GLAST/EAAT1 (C-terminus), and FITC-conjugated anti-GFP were obtained from Abcam (Cambridge, UK); mouse monoclonal anti-glial fibrillary acidic protein (GFAP) was obtained from Millipore; goat polyclonal anti-OSMR-β (AF662) was from R&D Systems (Minneapolis, USA); mouse monoclonal anti-MAP2 and mouse monoclonal anti-α-tubulin were obtained from Sigma-Aldrich; and primary antibodies against total and phosphorylated signal transducers and activators of transcription (STAT)3, extracellular signal-regulated kinase ½ (ERK1/2), and Akt were obtained from Cell Signaling Technology (Bioke, Leiden, The Netherlands). The fluorescent dye-conjugated secondary antibodies used for Western blot, donkey anti-mouse IR Dye 680, and donkey anti-rabbit IR Dye 800CW were obtained from LI-COR Biosciences (Cambridge, UK). The fluorescent dye-conjugated secondary antibodies used for immunocytochemistry: goat anti-rabbit CY3 was obtained from Jackson ImmunoResearch Laboratories (Uden, The Netherlands); and donkey anti-mouse Alexa Fluor 488 was obtained from Molecular Probes (Breda, The Netherlands).

### Animals

All procedures carried out were in strict accordance with recommendations in the Guide for Care and Use of Laboratory Animals of the National Institutes of Health, and the regulations of the Ethical Committee for the use of experimental animals of the University of Groningen, The Netherlands (License number DEC 4623A and DEC 5913A), Institutional Animal Care and Use Committee of the University of Minnesota (Protocol no: 1203A11091 and 1404A31457), as well as with the Portuguese law on Animal Care and European Union guidelines (Directive 2010/63/EU). Wild-type C57BL/6J (1–2 days postnatal) mice were obtained from Charles River and Central laboratory animal facility of Groningen. Wild-type C57BL/6J (14–15 days embryonic) mice were obtained from Harlan (Horst, The Netherlands). Animals were housed in standard makrolon cages and maintained on a 12-h light/dark cycle. They received food and water *ad libitum*.

### Primary astrocyte culture

Primary astrocyte cultures were established from cerebral cortices of postnatal (1–2 days) C57BL/6J mice according to a previously described procedure [41], modified to reduce microglial contamination [42]. Approximately 2 weeks after plating, microglial cells were mechanically separated from the astrocytic monolayer by shake-off at 150 rpm for 1 h. This procedure was repeated twice with an interval of 4 days in vitro between each shake-off, followed by an overnight shake-off at 240 rpm to remove oligodendrocyte precursor cells. Enriched astrocytes were washed with HBSS buffer (in mM: 137 NaCl, 5.3 KCl, 0.3 Na_2_HPO_4_, 0.4 KH_2_PO_4_, 4.2 NaHCO_3_, 5.6 d-glucose, pH 7.4) containing 1 mM ethylenediaminetetraacetic acid (EDTA) and further detached using 0.1 % trypsin (diluted in HBSS). Cells were reseeded with fresh astrocyte culture medium (DMEM supplemented with 5 % FCS, 2 mM l-glutamine, 1 mM sodium pyruvate, and 50 U/mL penicillin-streptomycin) in multiwell plates (5 × 10^4^ cells/cm^2^) and maintained in culture to confluence. To further reduce microglial contamination, confluent astrocyte cultures were treated with 5 mM LME, a lysosomotropic agent [43], for 4–5 h. Astrocytes were ready for experiments after 1–2 days. Our cell preparations had a high percentage of astrocytes (≥95 %), which was confirmed by immunostaining against GFAP (astrocyte marker) and CD11b (microglial marker) (Additional file 1: Figure S1) [44]. Although with low abundance, it is possible that the presence of microglial cells (approx. 5 %) in astrocyte cultures may influence some of the observations in this study. Therefore, additional experiments were carried out to verify astrocyte specificity of OSM effects in astrocyte cultures treated with liposomal clodronate (1 mg/mL for 4 h; Encapsula NanoSciences LLC, TN, USA), which resulted in complete depletion of microglial cells (Additional file 2: Figure S2A).

### BV2 cell line and primary microglia culture

The murine microglial BV2 cells were maintained in DMEM media containing 10 % FCS at 37 °C in a 5 % CO_2_ incubator. Primary microglia cultures were established following their shake-off (150 rpm for 1 h) from mixed glial culture flasks. The cells were cultured in standard DMEM media (supplemented with 5 % FCS, 2 mM l-glutamine, 1 mM sodium pyruvate and 50 U/mL penicillin-streptomycin), diluted in 1:1 ratio with glial-conditioned media.

### Primary neuronal culture

Primary cultures of cortical neurons from mouse embryo (~E_15_) were done as described previously [26]. Briefly, cortices from embryonic brains were dissected in ice-cold HBSS supplemented with 30 % glucose. Meninges were removed, and the tissues were treated with trypsin before they were gently dissociated by trituration in neuronal culture media (neurobasal medium supplemented with 2 % B27, 1 mM sodium pyruvate, 2 mM l-glutamine and 50 U/mL penicillin-streptomycin). Cell suspension was filtered using a cell strainer (70 μm; Falcon, Franklin Lakes, NJ, USA) before centrifugation (800 rpm for 10 min). Cells were then seeded on poly-d-lysine (10 μg/mL)-coated 96-well plates (1 × 10^5^ cells/well) and maintained in neuronal culture media in a humidified atmosphere with 5 % CO_2_ at 37 °C. Culture media was refreshed 24 h later to minimize culture debris. The neuronal purity as determined by MAP2 staining was around 98 % [26]. Cultures were used after 5 days in vitro.

### EcoHIV infection

In the present work, we used EcoHIV/NL4-3-EGFP (referred to as EcoHIV, for brevity), a chimeric HIV-1 expressing enhanced green fluorescent protein (EGFP) as an indicator, which was constructed on the backbone of HIV-1/NL4-3, as described previously [39]. Infectious EcoHIV stocks were propagated in HEK293TN cells as described [40] and titered for the p24 HIV-1 core antigen by ELISA, following the manufacturer’s instructions (ZeptoMetrix Corporation, NY, USA). Cultured astrocytes, microglia, and BV2 cells were incubated with 35,000 pg of p24 (per 1 × 10^6^ cells) during the incubation time stated in the text. Viral infectivity of cells was assessed by HIV LTR (long-term repeat) gene expression and/or anti-GFP immunocytochemistry.

### RNA isolation and reverse transcription polymerase chain reaction (RT-PCR)

Cultured cells were lysed in guanidinium isothiocyanate/mono-thioglycerol (GTC^+^) buffer and total RNA was extracted using a phenol-chloroform/iso-amyl alcohol step, followed by DNAse 1 treatment. Purified messenger ribonucleic acid (mRNA) was then transcribed into complementary DNA (cDNA) as described previously [45]. The quality of the cDNA was examined using primers for the housekeeping gene glyceraldehyde-3-phosphate dehydrogenase (GAPDH; see Table 1). Potential contamination of mRNA samples by genomic deoxyribonucleic acid (DNA) was checked by running reactions without reverse transcriptase, using GAPDH primers for the subsequent PCR amplification.

**Table 1:** Primers used for reverse transcription polymerase chain reaction (RT-PCR)

Gene	Forward primer (5′-3′)	Backward primer (5′-3′)
GAPDH	CATCCTGCACCACCAACTGCTTAG	GCCTGCTTCACCACCTTCTTGATG
OSMR-β	ATTCTGGACACGAAGAGGTCAAG	TTCCACTGCAAATCACAGCG
gp130	TGGAAGGCACTGCCTCTTTC	CTAGAGACGCGACATAGCGGT
GLAST	CCTTCGTTCTGCTCACGGTC	TTCACCTCCCGGTAGCTCAT
GLT-1	GTGCAAGCCTGTTTCCAGC	GCCTTGGTGGTATTGGCCT

### Real-time polymerase chain reaction (qPCR)

The expression of OSM, OSMR-β, gp130, GLAST, GLT-1, and HIV LTR genes was analyzed by real-time PCR using an iCycler (Bio-Rad, Veenendaal, The Netherlands) and iQ SYBR Green supermix (Bio-Rad). The housekeeping gene, hypoxanthine phosphoribosyl transferase 1 (HPRT1), was used for normalization, and it showed no variations in response to the experimental treatment (see Table 2 for primer sequences). The comparative *C*_*t*_ method (amount of target amplicon *X* in sample *S*, normalized to a reference *R* and related to a control sample *C*, calculated by 2 − ((*C*_*t*_*X*,*S* − *C*_*t*_*R*,*S*) − (*C*_*t*_*X*,*C* − *C*_*t*_*R*,*C*)) was used to determine the relative expression levels of all tested genes [46]. Linear regression analysis of the data was performed to understand the effect of OSM treatment over time on the expression of GLAST and GLT-1 mRNA.

**Table 2:** Primers used for real-time polymerase chain reaction (qPCR)

Gene	Forward primer (5′-3′)	Backward primer (5′-3′)
GAPDH	ATGGCCTTCCGTGTTCCTAC	GCCTGCTTCACCACCTTCTT
HPRT1	GACTTGCTCGAGATGTCA	TGTAATCCAGCAGGTCAG
GLAST	CCTTCGTTCTGCTCACGGTC	TTCACCTCCCGGTAGCTCAT
GLT-1	GTGCAAGCCTGTTTCCAGC	GCCTTGGTGGTATTGGCCT
OSM	GTGGCTGCTCAACTCTTCC	AGAGTGATTCTGTGTTCCCCGT
OSMR-β	ATTCTGGACACGAAGAGGTCAAG	TTCCACTGCAAATCACAGCG
gp130	TGGAAGGCACTGCCTCTTTC	CTAGAGACGCGACATAGCGGT
HIV LTR	GGTCTCTCTGGTTAGACCAGAT	CTGCTAGAGATTTTCCACACTG
IL-1β	GGCAGGCAGTATCACTCATT	AAGGTGCTCATGTCCTCATC
TNF-α	GACGTGGAACTGGCAGAAGA	GCCACAAGCAGGAATGAGAA
COX-2	CTCCCTGAAGCCGTACACAT	CCCAAAGATAGCATCTGGA
iNOS	AAGGCCACATCGGATTTCAC	GATGGACCCCAAGCAATACTT
GFAP	GTTTCATCTTGGAGCTTCTGC	GGAGGTGGAGAGGGACAAC
IL-6	CCGGAGAGGAGACTTCACAG	TCCACGATTTCCCAGAGAAC

### Western blotting

Western blotting of primary astrocyte and microglia cell lysates was performed as previously described [26]. Protein calibration controls were performed to ensure that the chosen working quantities were at non-saturating conditions (Additional file 3: Figure S3B-C). Briefly, equal amounts of protein (30 μg) were loaded onto 12.5 % sodium dodecyl sulfate-polyacrylamide gels and subsequently transferred to PVDF membranes. The membranes were blocked using Odyssey blocking buffer (OBB; diluted 1:1 in PBS) for 1 h and incubated overnight at 4 °C with different combinations of primary antibodies (diluted in 1:1 OBB and PBS-T (PBS + 0.1 % Tween 20)): goat anti-OSMR-β, rabbit anti-GLAST, rabbit anti-GLT-1, mouse anti-β-actin, mouse anti-α-tubulin, mouse anti-STAT3, mouse anti-p44/42 mitogen-activated protein kinase (MAPK), mouse anti-Akt (Pan), rabbit anti-phospho STAT3 (Tyr705), rabbit anti-phospho-p44/42 MAPK (Thr202/Tyr204), and rabbit anti-phospho-Akt (Ser473). The next day, membranes were washed in PBS-T (4 × 5 min) and incubated for 1 h at room temperature (with gentle shaking in the dark) with appropriate fluorescent dye-conjugated secondary antibodies (diluted in PBS-T): donkey anti-goat IR Dye 800CW, donkey anti-mouse IR Dye 680, and donkey anti-rabbit IR Dye 800CW. Membranes were washed again in PBS-T (4 × 5 min), and fluorescent bands were detected using LI-COR’s Odyssey infrared imaging system. The densitometry analysis of protein bands was performed using ImageJ software (NIH) [47].

### OSM and IL-6 ELISA

Supernatants from cultured BV2, primary shake-off microglia, and primary astrocytes were collected following EcoHIV infections, to measure secreted OSM levels using a mouse OSM ELISA-kit (USCN Life Science Inc., TX, USA) following the manufacturer’s instructions. Secreted IL-6 levels in astrocyte culture supernatants were measured using a mouse IL-6 ELISA Ready-SET-Go kit (Affymetrix, eBioscience, CA, USA), following the manufacturer’s instructions.

### Determination of astrocytic glutamate uptake: ^3^H-d-aspartate uptake assay

d-Aspartate was used as index of glutamate uptake in this study as it is a substrate for the high-affinity glutamate transporters with slow intracellular metabolization, thus allowing a more precise measure of glutamate transporter activity [48–50]. The analysis of ^3^H-d-aspartate uptake was evaluated as previously described [41]. Briefly, cultured astrocytes were incubated with Krebs buffer (in mM: 132 NaCl, 4 KCl, 1.2 Na_2_HPO_4_, 1.4 MgCl_2_, 6 glucose, 10 HEPES, 1 CaCl_2_, pH 7.4) containing ^3^H-d-aspartate (0.1 μCi/mL) and 50 μM d-aspartate for 10 min at 37 °C. Subsequently, the medium was removed and the cultured cells were placed on ice and washed twice with cold NMG buffer (where NaCl is replaced by *N-*methylglucamine, NMG) to terminate the uptake process. The cells were lysed with 0.5 M NaOH and transferred to a scintillation vial to be mixed with liquid scintillation cocktail. The radioactivity content (disintegrations per minute) was determined using liquid scintillation counting on a TRICARB® 2900TR analyzer. The remaining cell suspension was used to determine the protein content with the bicinchoninic acid (BCA) method (Pierce Technology) [51]. The uptake rate was expressed as the uptake per minute per milligram of protein. For saturation kinetics assays, the total d-aspartate concentrations ranged from 5 to 200 μM. The kinetic constants (i.e., maximum velocity, *V*_max_, and Michaelis-Menten constant, *K*_*M*_) were determined by using nonlinear regression fit of the data with a rectangular hyperbola, using the GraphPad Prism software (version 5.02, GraphPad Software Inc, La Jolla, CA, USA).

### Induction of excitotoxicity

Confluent astrocyte cultures were treated without or with OSM (10 ng/mL for 24 h) in the presence or absence of STAT3 activation inhibitor AG490 (25 μM, added 2 h before OSM). The cultures were then washed with warm HBSS and incubated with glutamate (100 μM, diluted in neuronal culture media) at 37 °C for 30 min. The supernatants from astrocytes incubated with glutamate, hereinafter referred to as glutamate^inc.^, were collected and applied (diluted 1:1 with neuronal media) onto 5 days old embryonic cortical neuron cultures and incubated for 1 h at 37 °C. Some neuron cultures were also treated with 50 μM glutamate for 1 h (positive control for excitotoxicity). Where indicated, neurons were pre-incubated with the NMDA receptor antagonist, dizocilpine (MK 801; 30 μM) for 30 min before the glutamate or the glutamate^inc.^ treatment. Following glutamate treatment, the neuronal medium was refreshed and cultures were incubated for the indicated period of time before they were assessed for cellular dysfunction and cytotoxicity using MTT assay and propidium iodide labeling, respectively.

### Determination of neuronal viability

1. * MTT assay*: The metabolic viability of cultured embryonic cortical neurons was measured 24 h after glutamate treatment by the colorimetric MTT (3-(4,5-dimethylthiazol-2-yl-) 2,5-diphenyltetrazolium bromide) assay, as described previously [26, 52]. MTT solution (0.5 mg/mL final concentration) was added to cultured neurons and incubated for 4 h. Following incubation, the cells were lysed and MTT-formazan solubilized in dimethyl sulfoxide (DMSO) with an orbital shaker for 15 min. The optical density of each sample was determined using an automated ELISA reader (Varioskan Flash spectral scanning multimode reader; Thermo scientific, USA) at 570 nm, with a background correction at 630 nm.
2. *Propidium iodide labeling*: Survival of cultured cortical neurons was estimated using propidium iodide (PI; DNA intercalating dye) labeling gauged by immunocytochemistry, as described previously [26]. Briefly, neuronal cultures treated without or with glutamate (or glutamate^inc.^) were incubated with PI (5 μg/mL; directly added to culture media) for 4 h and fixed in 4 % paraformaldehyde. After a few washes with PBS, cells were blocked using 5 % normal goat serum (NGS) diluted in PBS^+^ (PBS containing 0.1 % Triton-X100) for 1 h at room temperature on a shaker and subsequently stained with a mouse anti-MAP2 primary antibody (1:600; diluted in PBS^+^ with 1 % NGS) and incubated overnight at 4 °C on a shaker. Following primary antibody incubation, cells were washed with PBS (4 × 5 min) and incubated with the donkey anti-mouse secondary Alexa Fluor 488-conjugated antibody (1:400; diluted in PBS^+^) for 1 h at room temperature in the dark on an orbital shaker. A counterstain with Hoechst 33342 (1:1000; diluted in PBS) was performed to detect cell nuclei (not shown), and fluorescent signals were analyzed by confocal imaging using a Leica SP2 AOBS system (Leica Microsystems, Heidelberg, Germany).

### Statistical data analysis

The absolute data values were normalized to control in order to allow multiple comparisons. Statistical analyses were performed by one-way analysis of variance (ANOVA) followed by Bonferroni and Dunnett post hoc tests, using the Statistical Package for the Social Sciences (SPSS, Chicago, IL, USA) and GraphPad Prism software (version 5.02, GraphPad Software Inc, La Jolla, CA, USA). In all cases, *p* values <0.05 were considered statistically significant.

## Results

### OSM down-regulates GLT-1 and GLAST expression in primary mouse cortical astrocytes

**Fig. 1:**
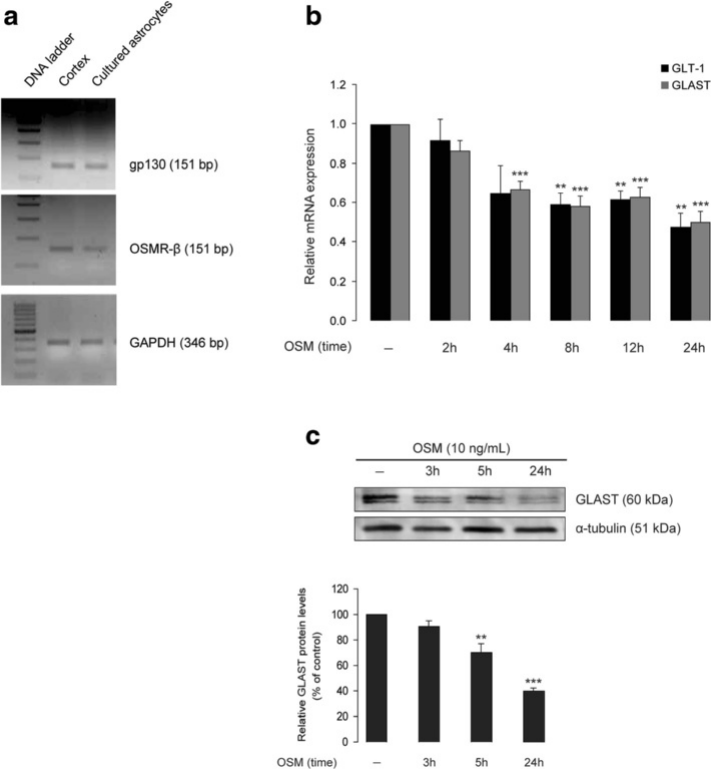
OSM down-regulates GLT-1 and GLAST expression in primary mouse cortical astrocytes. **a** Total mRNA purified from wild-type (C57BL/6J) mouse neonatal (P2) brain cortex as well as from cultured cortical astrocytes established from P2 brains was analyzed for expression of OSMR-β and gp130 mRNA by reverse transcriptase PCR; GAPDH primers were used as loading control. **b** Cortical astrocyte cultures were treated without or with OSM (10 ng/mL) for 2, 4, 8, 12, and 24 h and analyzed for GLT-1 and GLAST mRNA levels (gene expression normalized to HPRT1) by real-time PCR. Data are normalized to untreated controls and presented as mean ± SEM; *n* = 3, **p < 0.01, ***p < 0.001; one-way ANOVA using Bonferroni correction. **c** Cortical astrocytic cultures were treated without or with OSM (10 ng/mL) for 3, 5, and 24 h and were analyzed for GLAST proteins by Western blot. Relative densitometric analysis of GLAST proteins is shown in the *lower panel*. Data are presented as percentage of each respective ratio between optical density value of GLAST band intensity and optical density value of the matched α-tubulin (which served as the loading control) band intensity; *n* = 3, **p < 0.01, ***p < 0.001; one-way ANOVA

Existing literature demonstrates that, in mice, OSM acts on target cells through a receptor complex consisting of a ligand recognition subunit (OSMR-β) and a signal transducing subunit (gp130) [53, 54]. Furthermore, all receptor components required for OSM signaling are expressed in human astrocytes [36]. In the present work, we first confirmed expression of gp130 and OSMR-β mRNA in cultured cortical astrocytes established from neonatal (P2) mouse brains (Fig. 1a). We next investigated whether activation of OSM receptors in cortical astrocytes regulates expression of GLT-1 and GLAST genes in vitro. Cultured astrocytes treated with recombinant mouse OSM (10 ng/mL) for different time periods (2, 4, 8, 12, and 24 h) were analyzed for GLT-1 and GLAST mRNA expression by real-time PCR (Fig. 1b). Linear regression analysis showed a time-dependent reduction of the expression of GLT-1 (*R*^2^ = 0.415, *p* = 0.001, *n* = 3) and GLAST (*R*^2^ = 0.525, *p* < 0.001, *n* = 3) mRNA in OSM-treated astrocytes compared to the control; this effect was apparent after 4 and 8 h of OSM incubation, for GLAST and GLT-1, respectively (Fig. 1b). In order to understand whether the 5 % microglial cells present in astrocyte cultures influence this effect of OSM, we depleted microglial cells with liposomal clodronate (1 mg/mL for 4 h) before addition of OSM (Additional file 2: Figure S2A-B). Treatment with OSM (10 ng/mL for 24 h) of microglia-depleted astrocyte cultures reduced both GLT-1 and GLAST mRNA (*p* < 0.001, *n* = 3) (Additional file 2: Figure S2B), and this effect of OSM was comparable to the observations made from astrocyte cultures containing 5 % microglia (Fig. 1b) further establishing that OSM receptors are primarily expressed in astrocytes.

It is well known that astrocyte cultures, due to the absence of soluble neuronal factors, express GLT-1 protein at very low levels [55, 56]. Consistently, in our study, GLT-1 protein levels in cultured cortical astrocytes were minimally detected by Western blot (Additional file 3: Figure S3C-D) and thus making them difficult to quantify. In contrast, GLAST proteins were robustly expressed in astrocyte cultures (Additional file 3: Figure S3B). Treatment with OSM (10 ng/mL) induced a time-dependent reduction of GLAST protein density (Fig. 1c), consistent with the reduction of GLAST mRNA levels (Fig. 1b). When compared to the untreated control, a 5-h OSM treatment significantly decreased of GLAST protein density (30.0 ± 7.0 %, *n* = 3, *p* < 0.01), while a 24-h OSM treatment further decreased GLAST protein density (60.1 ± 2.4 %, *n* = 3, *p* < 0.001) (Fig. 1c).

### OSM inhibits ^3^H-d-aspartate uptake in primary mouse cortical astrocytes

**Fig. 2:**
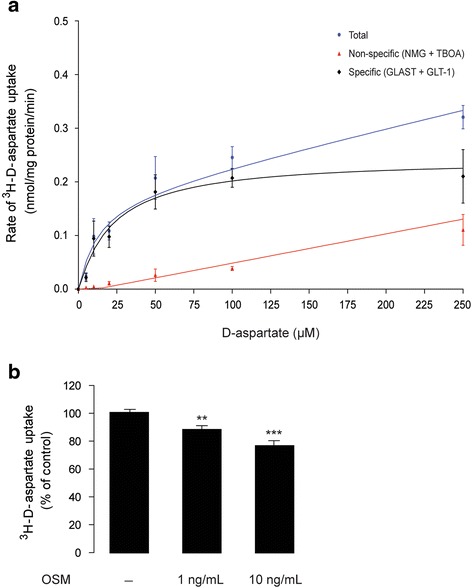
OSM inhibits d-aspartate uptake in primary mouse cortical astrocytes. **a** Shows saturation isotherm of Na^+^-dependent ^3^H-d-aspartate uptake by astrocytes. Primary astrocytes from wild-type (C57BL/6J) mouse neonatal (P2) brains were incubated with ^3^H-d-aspartate (0 to 200 μM) dissolved either in a Na^+^ buffer or in a Na^+^-free buffer (where Na^+^ is replaced by *N*-methyl-d-glucamine chloride, NMG, to determine nonspecific tritium retention) for 10 min at room temperature. Each point represents the mean ± SEM of at least three separate experiments, measured in triplicate. Kinetic constants were determined by nonlinear regression fit or a rectangular hyperbola, where *V*_max_ was 0.3 μM ± 0.08 and *K*_M_ was 22.4 μM (95 % confidence interval: 0.17–0.34 μM). **b** Shows the effect of OSM treatment (1 and 10 ng/mL; for 24 h) on ^3^H-d-aspartate uptake in cortical astrocytes culture. Data are normalized to untreated controls and presented as mean ± SEM; *p < 0.05, **p < 0.01, *n* = 3 to 17; one-way ANOVA followed by Dunnett’s multiple comparison test

We next investigated whether the down-regulation of GLT-1 (mRNA) and GLAST (mRNA and protein) by OSM had consequences on the ability of astrocytes to clear extracellular glutamate. The rate of ^3^H-d-aspartate uptake, as determined by the saturation isotherm, was maximal when astrocytes were incubated with 50 μM of ^3^H-d-aspartate for 10 min at room temperature (Fig. 2a). As shown in Fig. 2b, cultured astrocytes treated with OSM (1 or 10 ng/mL, for 24 h) displayed a concentration-dependent decrease of ^3^H-d-aspartate uptake (OSM 1 ng/mL: 12.2 ± 2.2 % decrease, *n* = 3, *p* < 0.05; OSM 10 ng/mL: 24 ± 4 % decrease, *n* = 3, *p* < 0.01).

### JAK/STAT3 inhibition prevents OSM-induced reduction of astrocytic ^3^H-d-aspartate uptake

**Fig. 3:**
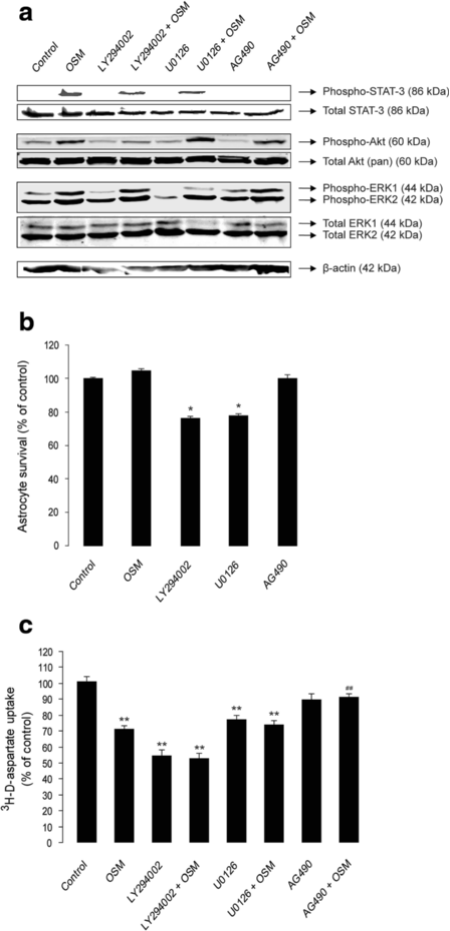
Inhibition of JAK/STAT3, but not PI3K/Akt or MEK/ERK1/2 signaling pathways, prevents OSM-induced reduction of ^3^H-d-aspartate uptake in primary cortical astrocytes. **a** Shows the effect of 2 h pre-treatment with selective inhibitors of PI3K (LY294002, 25 μM), MEK1/2 (U0126, 5 μM), and JAK (AG490, 25 μM) on activation of Akt, ERK1/2, or STAT3 proteins, respectively, in OSM-treated (10 ng/mL for 1 h) astrocytes culture. Activation of different signaling proteins was evaluated by Western blot using antibodies that selectively detect the phosphorylation at Ser473, Thr202/Tyr204, and Tyr705 residues of Akt, Erk1/2, and STAT3, respectively. Total amounts of each protein were detected using appropriate antibodies (see the “Methods” section); β-actin served as loading control. **b** Shows the effect of OSM (10 ng/mL), LY294002 (25 μM), U0126 (5 μM), and AG490 (25 μM) treatments for 24 h on the viability of cultured cortical astrocytes. Cell viability was measured using a colorimetric MTT assay. OD measurements were made at 570 nm, with a blank correction made at 630 nm. Data are presented as percentage of untreated (control) astrocytes; *each bar* represents the average of four independent experiments done in quadruplicates. *p < 0.01. **c** Shows the effect of pre-treatment with LY294002 (25 μM), U0126 (5 μM), and AG490 (25 μM) on ^3^H-d-aspartate uptake in untreated and OSM-treated (10 ng/mL, for 24 h) astrocyte cultures. Data are normalized to untreated controls and presented as mean ± SEM; **p < 0.001 (compared to untreated control); ^##^ *p* < 0.001 (compared to OSM); *n* = 8; one-way ANOVA

It has been previously shown that OSM binding to the OSMR-β/gp130 receptor complex results in phosphorylation of tyrosine residues of the gp130 receptor; this triggers the recruitment, phosphorylation, and activation of STAT proteins, mainly STAT1, STAT3, and STAT5 [57–59]. In addition, OSM activates other signaling pathways such as phosphatidylinositol 3-kinase (PI3K/Akt) [60] and the MAPK, namely, ERK1/2, p38, and c-jun N-terminal kinases/stress-activated protein kinases (JNK/SAPK) [61]. We next investigated whether treatment with OSM induced phosphorylation of the three principal downstream pathways: JAK/STAT3, PI3K/Akt, and ERK1/2-MAPK [35] in cultured astrocytes. As shown in Fig. 3a, basal activation of ERK1/2 and Akt proteins, but not STAT3, was observed in untreated (control) primary astrocytes. OSM treatment (10 ng/mL for 1 h) induced the phosphorylation at Tyr705, Ser473, and Thr202/Tyr204 residues of STAT3, Akt, and ERK1/2 proteins, respectively (Fig. 3a). Treatment of cultured astrocytes (2 h prior to OSM) with selective inhibitors of PI3K (LY294002, 25 μM), MEK1/2 (U0126, 5 μM), and JAK (AG490, 25 μM) inhibited OSM-induced phosphorylation of Akt, ERK1/2, and STAT3 proteins, respectively (Fig. 3a). In the subsequent experiments, we investigated how selective inhibition of PI3K/Akt, ERK1/2, and JAK/STAT3 signaling pathways regulated OSM-induced reduction of glutamate transporter activity in cultured astrocytes. Treatment with OSM or AG490 did not affect the survival of cultured astrocytes (Fig. 3b), whereas both LY294002 and U0126 induced about 20 % reduction (*p* < 0.01) in astrocyte survival (Fig. 3b). The blockade of Akt, ERK1/2, and STAT3 signaling pathways occluded the OSM-induced inhibition of ^3^H-d-aspartate uptake (Fig. 3c). However, whereas AG490 did not modify astrocytic ^3^H-d-aspartate uptake, both LY294002 and U0126 decreased per se the uptake of ^3^H-d-aspartate (Fig. 3c). Taken together, these results indicate that STAT3 activation is sufficient to mediate the ability of OSM to impair glutamate uptake in mouse cortical astrocytes.

### OSM-induced inhibition of astrocytic glutamate uptake promotes NMDA-mediated excitotoxicity in cortical neurons in vitro

**Fig. 4:**
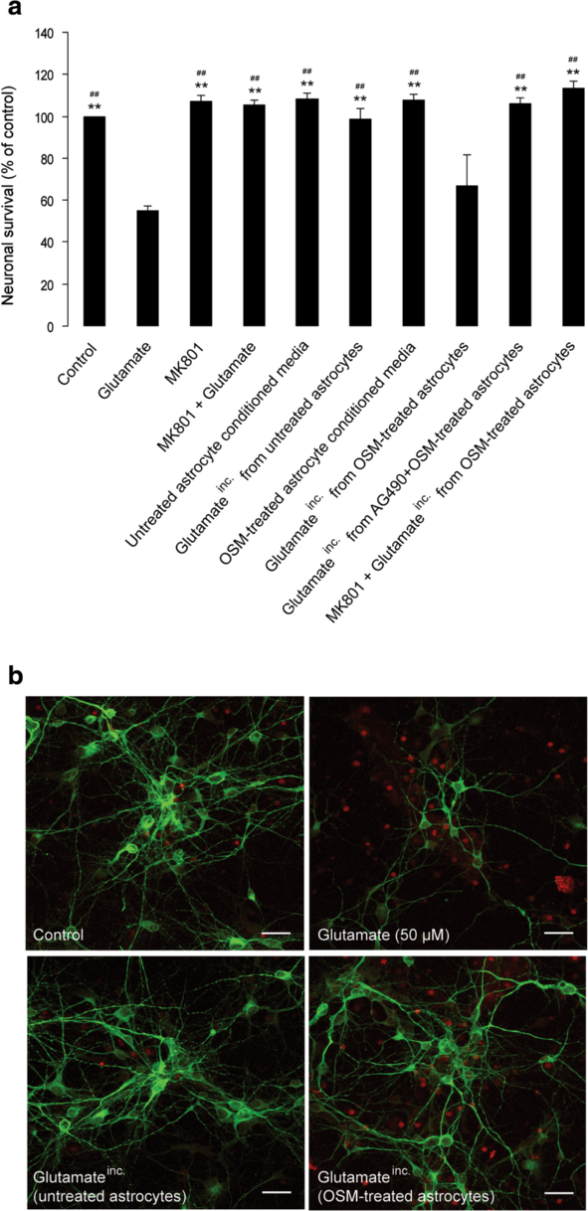
OSM-induced inhibition of astrocytic glutamate uptake promotes NMDA-mediated excitotoxicity in cortical neurons in vitro. Primary cortical astrocyte cultures from wild-type (C57BL/6J) mouse neonates (P2) were treated with OSM (10 ng/mL, for 24 h) in the absence or presence of a JAK/STAT3 inhibitor (AG490, 25 μM; added 2 h prior to OSM). Following OSM treatment, the cultures were washed once using warm HBSS and incubated with glutamate (100 μM, diluted in Neurobasal media) for 30 min. The resulting astrocyte supernatant, designated as glutamate^inc.^ (see the “Methods” section), was applied (diluted 1:1 ratio in neuronal culture media) to 6-day-old neuronal cultures obtained from embryonic (~E_15_) mouse cortex. As a positive control, neuronal cultures were treated with 50 μM glutamate, in the absence or presence of the NMDA receptor antagonist MK-801 (30 μM, added 30 min prior to glutamate treatment). Following glutamate treatment (for 1 h), new medium was added to the neuronal cultures and they were incubated at 37 °C in a CO_2_ incubator. **a** 24 h after glutamate treatment, cell viability was measured using a colorimetric MTT assay. OD measurements were made at 570 nm, with a blank correction made at 630 nm. Data are presented as percentage of untreated (control) neurons; *each bar* represents the average of four independent experiments done in quadruplicates. **p < 0.001 (compared to glutamate); ^##^ *p* < 0.001 (compared to glutamate^inc.^ from OSM-treated astrocytes); one-way ANOVA using Bonferroni correction. **b** 4 h after glutamate treatment, cells were fixed in 4 % paraformaldehyde, washed, and co-stained anti-MAP2 antibody (in *green*, neuronal marker) and propidium iodide (in *red*, showing cell death); *scale bar* corresponds to 25 μm

We next tested if this ability of OSM to inhibit astrocytic glutamate uptake translated into a detrimental effect of OSM on neurons. In order to investigate this, cultured astrocytes with or without OSM pre-treatment (10 ng/mL for 24 h) were incubated with glutamate (100 μM for 30 min). The glutamate^inc.^ (see Methods) was then applied to neuronal cultures. After 1 h of incubation at 37 °C, the medium was refreshed and neuronal viability was assessed after 4 and 24 h by immunocytochemistry and MTT assay, respectively (Fig. 4a, b). As a positive control, we used a direct addition of glutamate (50 μM) to neuronal cultures, which decreased neuronal viability by approximately 45 % (Fig. 4a) [26]. This glutamate-induced neuronal excitotoxicity was NMDA receptor-dependent since MK 801 (NMDA receptor antagonist; 30 μM) abolished the toxic effect of glutamate (*F* = 13.321; *p* < 0.001) (Fig. 4a). Finally, we observed that glutamate^inc.^ from OSM-treated astrocytes, but not untreated astrocytes, also reduced neuronal viability (OSM treated 67.1 ± 14.24 % survival, *n* = 4, *p* < 0.001 and untreated 98.4 ± 5.4 % survival, *n* = 4), when compared to the untreated control (Fig. 4a). These results were further confirmed by direct measure of neurotoxicity using PI staining (Fig. 4b). By contrast, neuronal viability was preserved when the cultures were co-treated with MK-801 (Fig. 4a), confirming that the neurotoxicity induced by glutamate^inc.^ from OSM-treated astrocytes was dependent on neuronal NMDA receptor activation. Since OSM-induced inhibition of glutamate uptake in cultured astrocytes was reversed by the JAK/STAT3 signaling inhibitor, AG490 (Fig. 3c), glutamate^inc.^ from cultured astrocytes that were pre-treated simultaneously with OSM and AG490 should not cause neuronal cell death. Indeed, neuronal viability was unchanged by glutamate^inc.^ from OSM-treated astrocytes where JAK/STAT3 activation was blocked (*F* = 13.321; *p* < 0.001) (Fig. 4a), reinforcing that OSM-induced inhibition of astrocytic glutamate transporter activity is regulated by the JAK/STAT3 signaling pathway.

### EcoHIV induces the expression and release of OSM in cultured BV2 cells and primary microglia

**Fig. 5:**
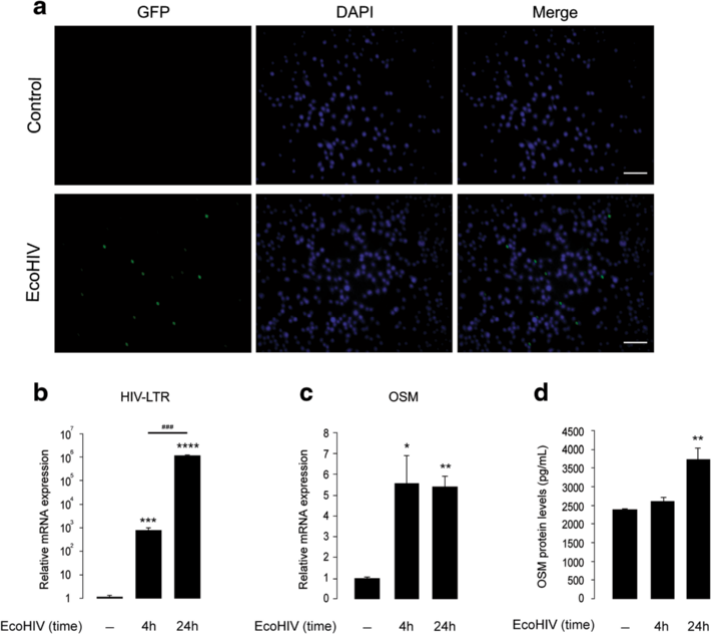
EcoHIV induces expression and release of OSM in cultured BV2 cells. **a** Shows GFP (in *green*, showing infected cells) and DAPI (in *blue*, showing nuclei) staining of control and EcoHIV-infected (35,000 pg of p24, for 4 h) BV2 cells; *scale bar* corresponds to 25 μm. **b**, **c** Show real-time PCR analyses of HIV LTR (**b**) and OSM (**c**) mRNA in control and EcoHIV-infected (35,000 pg of p24) BV2 cells. *p < 0.05, **p < 0.01, ***p < 0.001, ****p < 0.0001, ^###^ *p* < 0.001, *n* = 6. **d** Shows ELISA analysis of OSM proteins in culture supernatants collected from control and EcoHIV-infected (35,000 pg of p24) BV2 cells; **p < 0.01, *n* = 2 (in triplicates)

**Fig. 6:**
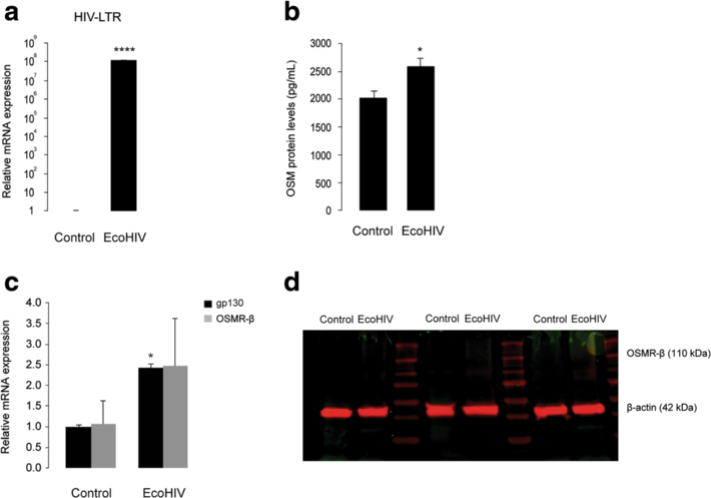
EcoHIV induces OSM release and gp130 mRNA, but not OSMR-β mRNA or protein, in cultured primary mouse microglia. **a** Shows real-time PCR analysis of HIV LTR mRNA in control and EcoHIV-infected (35,000 pg of p24, for 24 h) primary microglia. ****p < 0.0001, *n* = 3. **b** Shows ELISA analysis of secreted OSM proteins in culture supernatants collected from control and EcoHIV-infected primary microglia. *p < 0.05, *n* = 6. **c** Shows real-time PCR analysis of gp130 and OSMR-β mRNA in control and EcoHIV-infected primary microglia. *p < 0.05, *n* = 3. **d** Shows Western blot analysis for OSMR-β proteins in control and EcoHIV-infected primary microglial cell lysates obtained from three independent experiments. β-Actin served as the loading control

A recent study showed that PBMCs isolated from neurologically compromised HIV-1-infected patients spontaneously secreted high levels of OSM, whereas OSM levels secreted from PBMCs of neurologically asymptomatic HIV-1 patients were comparable to that of age-matched healthy subjects [20]. Furthermore, this OSM produced by PBMCs isolated from HIV-1-infected individuals induces toxicity in cultured primary human fetal neurons [29]. These findings strongly suggest an important role for OSM in neuropathogenesis and/or neurocognitive impairments observed in HIV-1-infected patients. However, it is not known if HIV-1 infection induces OSM production in microglial cells, a potential source for OSM in the CNS [18, 62]. In order to address this question, we next investigated the expression and release of OSM in cultured microglial cells that were infected with EcoHIV. The viral infection of BV2 cells for 4 h increased GFP-immunoreactivity (Fig. 5a), indicating successful viral infection. The viral infectivity of BV2 cells increased with time, as HIV LTR gene expression following 24 h of EcoHIV incubation was significantly higher when compared to that at 4 h of viral incubation (approx. 10^3^-fold increase at 4 h, *p* < 0.001; and 10^6^-fold increase at 24 h, *p* < 0.0001, *n* = 6) (Fig. 5b). Next, real-time PCR analysis of OSM gene in EcoHIV-infected BV2 cells showed a fivefold increase in expression of OSM mRNA both at 4 h (*p* < 0.05) and 24 h (*p* < 0.01) of viral incubation, when compared to uninfected controls (Fig. 5c). In addition, EcoHIV induced OSM release from BV2 cells, since the supernatants from infected cells (24 h following viral incubation) contained higher levels of secreted OSM (*n* = 2 in triplicates, *p* < 0.01), compared to the uninfected controls (Fig. 5d). We further confirmed the above findings in primary mouse microglia, as the culture supernatants contained elevated OSM levels (*n* = 6, *p* < 0.05) following 24 h of EcoHIV infection (Fig. 6a, b).

### EcoHIV induces gp130 mRNA, but not OSMR-β mRNA or protein, in cultured primary microglia

We next analyzed whether EcoHIV infection regulates expression of OSM receptor subunits (gp130 and OSMR-β) in cultured primary microglia. As shown in Fig. 6c, EcoHIV-infected microglia (following 24 h of treatment) showed a 2.4-fold increase in gp130 mRNA (*n* = 3, *p* < 0.05), when compared to the uninfected control. On the other hand, OSMR-β mRNA expression in primary microglia was not affected by EcoHIV infection (Fig. 6c). We further analyzed OSMR-β protein expression in primary microglia by Western blot. We observed that neither control nor EcoHIV-infected microglia express detectable levels of OSMR-β proteins (Fig. 6d). These observations are in line with a recent study that showed OSMR-β proteins are absent in microglial cells [63].

### EcoHIV does not affect OSM release but induces OSMR-β mRNA and protein expression in cultured cortical astrocytes

**Fig. 7:**
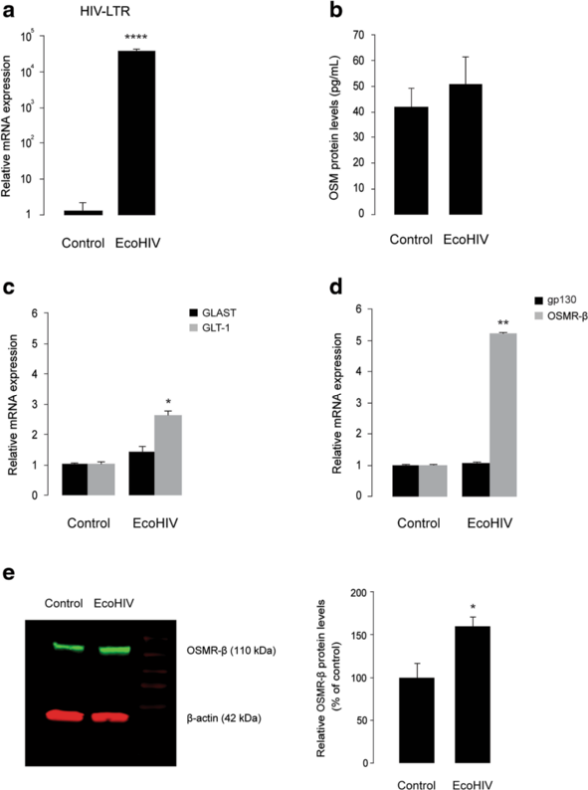
EcoHIV neither induces OSM secretion, nor the expression of gp130 or GLAST mRNA, but stimulates the expression of GLT-1 and OSMR-β in primary cortical astrocytes. **a** Shows real-time PCR analyses of HIV LTR mRNA in control and EcoHIV-infected (35,000 pg of p24, for 24 h) cortical astrocyte cultures. ****p < 0.0001, *n* = 3. **b** Shows ELISA analysis of secreted OSM proteins in culture supernatants collected from control and EcoHIV-infected primary astrocyte cultures. *n* = 6. **c**, **d** Shows real-time PCR analyses of GLAST and GLT-1 (**c**) and gp130 and OSMR-β (**d**) mRNA in control and EcoHIV-infected cortical astrocyte cultures. *p < 0.05, **p < 0.01, *n* = 3. **e** Shows Western blot analyses for OSMR-β proteins in control and EcoHIV-infected cortical astrocyte culture lysates. The *left panel* shows representative blot of three independent experiments. The *right panel* shows densitometric analysis of OSMR-β proteins. Data are presented as percentage of each respective ratio between optical density value of OSMR-β band (110 kDa) intensity and optical density value of the matched β-actin (42 kDa; loading control) band intensity. *p = 0.027, *n* = 3; two-tailed Student’s *t* test

Like microglia, astrocytes may also serve as a source for OSM in the CNS [18], since OSM immunoreactivity was detected in brains from MS patients in microglia, reactive astrocytes, and infiltrating leukocytes [18]. We next investigated whether EcoHIV induces OSM release from astrocytes. Cultured cortical astrocytes were infected for 24 h with EcoHIV, as confirmed by the HIV LTR gene expression (Fig. 7a), and culture supernatants were analyzed for secreted OSM levels. Control astrocytes secreted low levels of OSM (approximately 40 pg/mL) (Fig. 7b). However, in contrast to microglia, EcoHIV infection did not increase OSM secretion from cultured astrocytes (Fig. 7b).

It has been shown that HIV-1 directly inhibits glutamate transporter expression and glutamate uptake in human fetal astrocytes, without induction of pro-inflammatory mediators [64], and this effect was largely reproduced by treatment with the HIV-1 envelope protein gp120 alone [64]. The chimeric EcoHIV that we used in the present study does not contain gp120, as it is replaced with gp80 from murine leukemia virus [39, 40]. Thus, EcoHIV serve as a good model to investigate gp120-independent effects of HIV-1 in murine cells. We next investigated how EcoHIV infection regulated GLAST and GLT-1 genes in cultured cortical astrocytes. Interestingly, cultured astrocytes infected for 24 h with EcoHIV showed a 2.5-fold increase in GLT-1 mRNA (*n* = 3, *p* < 0.05) (Fig. 7c). By contrast, GLAST gene expression was unchanged in EcoHIV-infected astrocytes, when compared to the untreated control (Fig. 7c). We next investigated whether EcoHIV-infected astrocytes displayed an enhanced gene expression of OSM receptor subunits, OSMR-β, and gp130. As shown in Fig. 7d, EcoHIV infection did not affect the expression of gp130 mRNA in cultured astrocytes. On the other hand, EcoHIV-infected astrocytes showed a fivefold increase in the expression of OSMR-β mRNA (*n* = 3, *p* < 0.01), compared to the uninfected control (Fig. 7d). Enhanced astrocytic expression of OSMR-β following EcoHIV infection was also observed at the protein level (*n* = 3, *p* < 0.05) (Fig. 7e). Taken together, our findings suggest that HIV-1 may additionally inhibit astrocytic glutamate uptake by a gp120-independent mechanism that involves a combined alteration of GLT-1 expression and the induction of OSMR-β expression and signaling in astrocytes.

## Discussion

This is the first report to show that treatment with OSM, a member of the IL-6 cytokine family, reduces glutamate uptake in cultured cortical astrocytes and thereby promotes excitotoxic death of cortical neurons in vitro. This effect of OSM is mediated by the down-regulation of the two Na^+^-dependent glutamate transporters, GLAST, and GLT-1. As shown in Fig. 1b, OSM treatment (10 ng/mL) reduces the expression of GLAST and GLT-1 mRNA in a time-dependent manner. The down-regulation of GLAST by OSM was also confirmed at the protein level; however, the low signals obtained with the GLT-1 antibody in the Western blot analysis precluded any reliable analysis of the impact of OSM on GLT-1 protein levels in cultured astrocytes (Additional file 3: Figure S3C-D). Consistent with the down-regulation of glutamate transporter expression, OSM inhibited ^3^H-d-aspartate uptake by astrocytes in a concentration-dependent manner (Fig. 2b), which mostly involved the recruitment of the JAK/STAT3 pathway (Fig. 3c), rather than the PI3K or ERK1/2 pathways. This is in agreement with the previously reported ability of OSM to induce STAT3 phosphorylation in astrocytes [65], which we now confirmed (Fig. 3a). We further showed that down-regulation of glutamate transport in astrocytes by OSM decreased survival of cultured cortical neurons. As shown in Fig. 4a, glutamate^inc.^ (see the “Methods” section) from untreated astrocytes did not affect the survival of cortical neurons, suggesting that extracellular glutamate is rapidly taken up by astrocytes. However, glutamate^inc.^ from OSM-treated astrocytes did cause excitotoxicity in cultured cortical neurons (Fig. 4a, b). It has been previously shown that gp130-mediated STAT3 activation precedes reactive gliosis in mouse astrocytes [33], which might lead to nitric oxide-induced inflammatory death of neurons [66]. In our current in vitro study, conditioned media from OSM-treated astrocyte cultures did not affect neuronal survival (Fig. 4a), and OSM treatment did not induce the production of nitric oxide in astrocyte cultures (Additional file 4: Figure S4), thus excluding the possibility of indirect oxidative stress-induced neuronal damage. These observations are supported by a recent study, where the authors showed that nitric oxide synthase is not induced by OSM in primary astrocytes and microglia [63]. Importantly, glutamate^inc.^ from OSM-treated astrocytes did not affect survival of neurons in the absence of neuronal NMDA receptor activity (Fig. 4a), suggesting that the increased neurotoxicity results from the decreased glutamate uptake in OSM-treated astrocytes.

The regulation of extracellular glutamate by astrocytes is determined by the density and activity of both glutamate transporters and glutamine synthetase, the enzyme that converts glutamate to glutamine [49]. Whether or not OSM treatment regulates glutamine synthetase expression or activity has not been addressed in this study. On the other hand, we showed that OSM treatment induces the expression of GFAP, COX2, and OSMR-β, but not gp130, in cultured astrocytes (Additional file 5: Figure S5A-C). In line with our findings, OSM has been previously shown to induce pro-inflammatory factors such as GFAP and COX2, among others in astrocytes [30, 35]. In addition, gp130-mediated STAT3 activation in striatal astrocytes has been reported to be closely associated with neuronal damage in a 1-methyl-4-phenyl-1,2,3,6-tetrahydropyridine (MPTP) model of neurodegeneration in vivo [33]. Furthermore, OSM/gp130-mediated STAT3 activation has been shown to mediate methamphetamine-induced astrogliosis [32]. Based on these findings, we provide direct evidence that the activation of OSM receptor, triggering STAT3 signaling in astrocytes, impacts neuronal survival. Thus, blockade of STAT3 signaling in astrocytes might be beneficial to prevent excitotoxic neuronal death in models pertinent to many brain injuries with an inflammatory profile [33].

Astrocyte dysfunction resulting in deficient glutamate uptake and metabolism has been reported to be a major contributing factor to the excitotoxic death of neurons in different CNS disease conditions, including HAND [11, 12]. Thus, the identification of essential factors that regulate astrocytic glutamate transporter expression and activity might be beneficial in HAND treatment. It has been reported that HIV-1 could directly inhibit glutamate transporter expression and uptake in human fetal astrocytes, without induction of pro-inflammatory mediators such as TNF-α [64]. The authors of this study showed that the envelope protein, gp120, alone induced effects similar to these of HIV-1 [64]. In our study, we describe a potential gp120-independent mechanism for HIV-induced down-regulation of astrocytic glutamate transport using EcoHIV, a chimeric HIV-1 that can infect mouse cells [39, 40]. EcoHIV itself did not down-regulate GLAST expression in cultured mouse cortical astrocytes (Fig. 7c), whereas it enhanced GLT-1 mRNA expression by 2.5-fold. Interestingly, we show that EcoHIV infection induces a fivefold increase in OSMR-β mRNA and proteins in cultured astrocytes (Fig. 7d, e), whereas OSMR-β proteins in cultured microglia were undetectable by Western blot, as described previously [63]. We also provide evidence that EcoHIV infection induces OSM mRNA expression and protein release in BV2 cells and primary microglia (Figs. 5 and 6), but not in cultured astrocytes (Fig. 7b). In addition, it is noteworthy that the secreted OSM levels in untreated microglial culture supernatants were drastically higher, when compared to that of astrocytic culture supernatants (approximately 2000 and 40 pg/mL, respectively) (Figs. 6b and 7b), suggesting that microglial cells might be a better source for OSM release in the CNS [18, 62]. Further analysis using astrocyte-selective OSMR-β deficient animals or cell cultures would provide better insight into the role of OSM in EcoHIV-mediated neuropathogenesis and/or impaired astrocytic glutamate uptake. Several other pro-inflammatory mediators have been shown to regulate GLAST and GLT-1 expression in astrocytes, including TNF-α and IL-1β [67]. Real-time PCR analysis of TNF-α, IL-1β, cyclooxygenase-2 (COX-2), iNOS, and IL-6 genes in primary microglia showed an induction of these genes following EcoHIV infection (Additional file 6: Figure S6), indicating the complexity of the inflammatory processes that lead to impaired astrocytic glutamate uptake associated with EcoHIV infection.

Comparison of the findings reported in the present study with previous reports suggests that OSM has a complex profile of action in the control of neurodegeneration. In fact, previous studies demonstrated anti-inflammatory as well as neuroprotective properties of OSM, both in vitro and in vivo. For example, OSM inhibits production of pro-inflammatory mediators such as TNF-α, granulocyte macrophage colony-stimulating factor (GM-CSF), and IL-8 [24, 25] and has been shown to suppress inflammatory processes associated with the murine experimental allergic encephalomyelitis model of MS [24]. In addition, we have previously provided evidence for a neuroprotective effect of OSM against glutamate by up-regulating neuromodulatory adenosine A_1_ receptors [26]. In another study, direct activation of neuronal OSM receptors down-regulated the NR2C subunit of NMDA receptors and thereby prevented NMDA-induced toxicity [27]. More recently, the complex role of OSM signaling was further demonstrated by the reported neuroprotective activity of OSM against ischemic stroke, which is dependent on neuronal OSMR-β expression and activation, with decreased neuronal OSMR-β expression leading to worse stroke outcomes [28]. Taken together these findings and our present study, it may be concluded that the target cell addressed by OSM largely determines the pro- and anti-excitotoxic effects of this cytokine in the CNS.

In the mammalian brain, astrocytes are the predominant players in regulating the glutamate diffusion and spill over from perisynaptic areas, a pre-requisite process to maintain the high signal-to-noise ratio for synaptic communication [6, 49]. Therefore, compromised astrocytic glutamate uptake function caused by the overproduction of cytokines such as OSM, concomitant or resulting from brain injury, might synergistically exacerbate the accumulation of extracellular glutamate at excitotoxic concentrations leading to neuronal damage. In spite of their various suggested roles in astrocytic metabolism, IL-6 family members such as OSM have been scantily explored in their effects on glutamate uptake. There is evidence that CNTF, in contrast to our present findings with OSM, enhances both expression and activity of GLT-1 in astrocytes [68] and thereby promotes survival of neurons against excitotoxicity [69]. Several reports suggest an induction of IL-6 in astrocytes by variety of HIV proteins such as Tat, gp120, Nef, and Vpr [70–73]. Consistently, we show here that infection with EcoHIV virus for 24 h induces several-fold increase in IL-6 secretion in primary astrocytes (Additional file 7: Figure S7). However, IL-6 was shown to have no effect on glutamate uptake on cultured murine astrocytes [74, 75], although it suppressed the increased glutamate uptake induced by prostaglandin E2 (PGE2) [74]. Our preliminary findings show that IL-6 treatment (10 ng/mL for 24 h) did not significantly reduce GLT-1 mRNA expression in cultured astrocytes (*p* = 0.08, *n* = 5) (Additional file 8: Figure S8). On the other hand, we observed approximately 20 % reduction of GLAST mRNA (*p* = 0.04, *n* = 5) in IL-6-treated astrocyte culture (Additional file 8: Figure S8). However, this effect of IL-6 is mild, compared to the effect of OSM (10 ng/mL for 24 h) on GLAST gene expression in astrocytes (Fig. 1b and Additional file 2: Figure S2B), and requires further validation at the protein level. Taken together, in this study, we have shown that OSM, through STAT3 activation, impairs the capacity of astrocytes to remove glutamate from extracellular space, which may contribute to excitotoxic neuronal damage. This indicates that a better understanding of OSM signaling mechanisms regulating glutamate transporter level and activity may have important implications for developing novel strategies to limit excitotoxic brain damage in acute and neurodegenerative pathologies.

## Conclusions

OSM is a pleiotropic cytokine belonging to the IL-6 family that may differentially affect neuroinflammatory processes associated with disease conditions of the CNS. We have previously shown that OSM protects cultured neurons against glutamate-induced excitotoxicity, whereas others have reported that OSM mediates HIV-1-associated neurotoxicity, although the underlying mechanisms are unclear. Here, we provide the first evidence that OSM inhibits glutamate uptake process in astrocytes and thereby mediates neuronal excitotoxicity. We further demonstrate that a chimeric HIV-1 virus that infects murine cells (EcoHIV) induces expression and release of OSM in microglia and its receptor (OSMR-β) in astrocytes. Taken together, our findings suggest that targeting OSMR-β signaling in astrocytes might alleviate HIV-1-associated neuronal excitotoxicity.

## Abbreviations

ALS, amyotrophic lateral sclerosis; ANOVA, analysis of variance; BCA, bicinchoninic acid; CNS, central nervous system; COX-2, cyclooxygenase-2; DMEM, Dulbecco’s modified Eagle medium; DMSO, dimethyl sulfoxide; DNA, deoxyribonucleic acid; EAAT, excitatory amino acid transporter; EcoHIV, EcoHIV/NL4-3-GFP virus; EDTA, ethylenediaminetetraacetic acid; ERK1/2, extracellular signal-regulated kinase ½; FCS, fetal calf serum; GAPDH, glyceraldehyde-3-phosphate dehydrogenase; GFAP, glial fibrillary acidic protein; GLAST, glutamate aspartate transporter; GLT-1, glutamate transporter-1; GM-CSF, granulocyte macrophage colony-stimulating factor; gp130, glycoprotein 130; GTC, guanidinium isothiocyanate; HAND, HIV-associated neurocognitive disorders; HBSS, Hank’s buffered salt solution; HEPES, 4-(2-hydroxyethyl)-1-piperazineethanesulfonic acid; HPRT1, hypoxanthine phosphoribosyltransferase 1; IL, interleukin; JAK, Janus kinases; JNK, c-jun N-terminal kinases; LME, l-leucine methyl ester; MAP-2, Microtubule associated protein 2; MAPK, mitogen-activated protein kinase; MPTP, 1-methyl-4-phenyl-1,2,3,6-tetrahydropyridine; mRNA, messenger ribonucleic acid; MTT, 3-(4,5-dimethylthiazol-2-yl-) 2,5-diphenyltetrazolium bromide; NGS, normal goat serum; NMDA, *N*-methyl-d-aspartic acid; NMG, *N*-methyl-d-glucamine; OBB, Odyssey blocking buffer; OSM, oncostatin M; OSMR, oncostatin M receptor; PBS, phosphate-buffered saline; PCR, polymerase chain reaction; PGE2, prostaglandin E2; PI, propidium iodide; PI3K, phosphatidylinositol 3-kinase; PVDF, polyvinylidene fluoride; SAPK, stress-activated protein kinases; STAT, signal transducers and activators of transcription; TBOA, DL-threo-β-benzoyloxyaspartate; TNF, tumor necrosis factor

## Additional files

Additional file 1: Figure S1. Representative image (on top) showing the characteristic ratio of astrocytes-to-microglia in the cortical astrocytes culture. The cells were fixed in 4 % paraformaldehyde, immunostained for GFAP (astrocytes; in green) and CD11b (microglia; in red) and observed under a fluorescence microscope with a total magnification of ×1000. The graph below shows the average astrocyte cell number (~152) compared with the average microglial cell number (~10) after counting 10 microscopic fields in several 16 mm coverslips of astrocyte cultures in 4 independent experiments. The astrocyte purity was determined to be between 94 and 96 %. Scale bar corresponds to 20 μm. (TIF 16116 kb) Additional file 2: Figure S2. OSM down-regulates GLT-1 and GLAST expression in microglia-depleted primary mouse cortical astrocytes. (A) Representative image showing the purity of cultured astrocytes following treatment with liposome clodronate (1 mg/mL for 4 h). The cells were washed with PBS, fixed in 4 % paraformaldehyde, immunostained for GFAP (astrocytes; in green) and CD11b (microglia; in red) and observed under a fluorescence microscope with a total magnification of ×1000. Scale bar corresponds to 20 μm. (B) Shows real-time PCR analyses of GLT-1 and GLAST mRNA (gene expression normalized to HPRT1) in control and OSM-treated (10 ng/mL for 24 h) astrocyte cultures. The cultures used in this experiment were treated with liposomal clodronate for 4 h, washed three times with PBS, and incubated in fresh culture media for 24 h, before addition of OSM. Data are normalized to untreated controls and presented as mean ± SEM; ***p* < 0.01, *n* = 3. (TIF 8798 kb) Additional file 3: Figure S3. Primary mouse astrocyte cultures differentially express GLAST and GLT-1 transporters. (A) Cortical astrocyte cultures were established from wild-type (C57BL/6J) mouse neonatal (P2) brains, and total RNA was isolated and analyzed for the expression of GLAST (91 bp) and GLT-1 (86 bp) mRNA by reverse transcriptase PCR (see Table 1 for primer details); GAPDH (not shown) served as the loading control. (B, C) Protein lysates prepared from cultured astrocytes were analyzed for GLAST (B) and GLT-1 (C) protein levels by Western blot. α-Tubulin served as the loading control. The observation that GLT-1 proteins were only faintly detected when high amounts of protein were loaded is consistent with the previously published findings that showed GLT-1 protein in astrocyte cultures is expressed at almost undetectable levels [55, 56]. (D) Shows Western blot analysis of GLT-1 proteins in lysates prepared from adult mouse cortex (12-week-old), neonatal (P2) brain regions (hippocampus, olfactory bulb, cerebellum and cortex), and cultured cortical astrocytes obtained from P2 mouse brains. As shown, GLT-1 proteins were abundantly expressed in the adult mouse cortex, whereas their expression level in different brain regions of P2 mouse brain was very low. β-Actin served as the loading control. (TIF 14907 kb) Additional file 4: Figure S4. OSM treatment does not induce nitric oxide (NO) release in cultured cortical astrocytes. Cortical astrocyte cultures from wild-type (C57BL/6J) mouse neonatal (P2) brains were treated without or with OSM (10 ng/mL) for 2, 4, 8, 12, and 24 h. Following OSM incubation, the supernatants were directly analyzed for NO content using Griess reagent system as per manufacturer’s protocol. Data represent absolute values of NO concentration and are mean ± SEM of two independent experiments performed in triplicates. (TIF 6742 kb) Additional file 5: Figure S5. OSM treatment induces GFAP, OSMR-β, and COX-2 gene expression in cultured cortical astrocytes. (A) Shows real-time PCR analysis of GFAP mRNA in primary astrocyte cultures that were treated without or with OSM (10 ng/mL) for 2, 4, 8, 12, and 24 h. GFAP gene expression was transiently induced by OSM, peaking at 8–12 h of treatment. **p* < 0.05, *n* = 3. (B) Shows enhanced GFAP (green) immunoreactivity in cultured astrocytes following 8 h of OSM treatment (10 ng/mL), compared to the untreated control. Scale bar corresponds to 20 μm. (C) Shows real-time PCR analysis of OSMR-β, gp130, and COX-2 mRNA in primary astrocyte cultures that were treated without or with OSM (10 ng/mL) for 24 h. As shown, OSM induced two- and fivefold increase in OSMR-β and COX-2 mRNA, respectively. **p* < 0.05, ***p* < 0.01, *n* = 3. (TIF 19046 kb) Additional file 6: Figure S6. EcoHIV infection induces the expression of pro-inflammatory genes in primary microglia. Real-time PCR analysis of TNF-α (A), IL-1β (B), COX-2 (C), iNOS (D), and IL-6 (E) mRNA were performed in cultured primary microglia that were infected without (control) or with EcoHIV (35,000 pg of p24, for 24 h). As shown, infection with EcoHIV induced approximately 5-, 45-, 11-, 350-, and 360-fold increase in TNF-α, IL-1β, COX-2, iNOS, and IL-6 mRNA, respectively. **p* < 0.05, ***p* < 0.01, ****p* < 0.001, *****p* < 0.0001, *n* = 3. (TIF 23465 kb) Additional file 7: Figure S7. EcoHIV induces IL-6 release in cultured primary mouse astrocytes. Secreted IL-6 proteins in culture supernatants from control and EcoHIV-infected (35,000 pg of p24, for 24 h) primary astrocytes were measured using a mouse IL-6 ELISA Ready-SET-Go kit (Affymetrix, eBioscience), following the manufacturer’s instructions. *****p* < 0.0001, *n* = 6. (TIF 4562 kb) Additional file 8: Figure S8. IL-6 down-regulates GLAST, but not GLT-1, mRNA in primary mouse cortical astrocytes. Graph shows real-time PCR analyses of GLT-1 and GLAST mRNA (gene expression normalized to HPRT1) in control and IL-6-treated (10 ng/mL for 24 h) astrocyte cultures. Data are normalized to untreated controls and presented as mean ± SEM. **p* = 0.04 (GLAST); *p* = 0.08 (GLT-1); *n* = 5; two-tailed Student’s *t* test. (TIF 6507 kb)
