# Supplementary figures and images for: Oncostatin M promotes excitotoxicity by inhibiting glutamate uptake in astrocytes: implications in HIV-associated neurotoxicity
Source: J Neuroinflammation. 2016 Jun 10;13:144. doi: 10.1186/s12974-016-0613-8 (PMC4903004; doi:10.1186/s12974-016-0613-8)

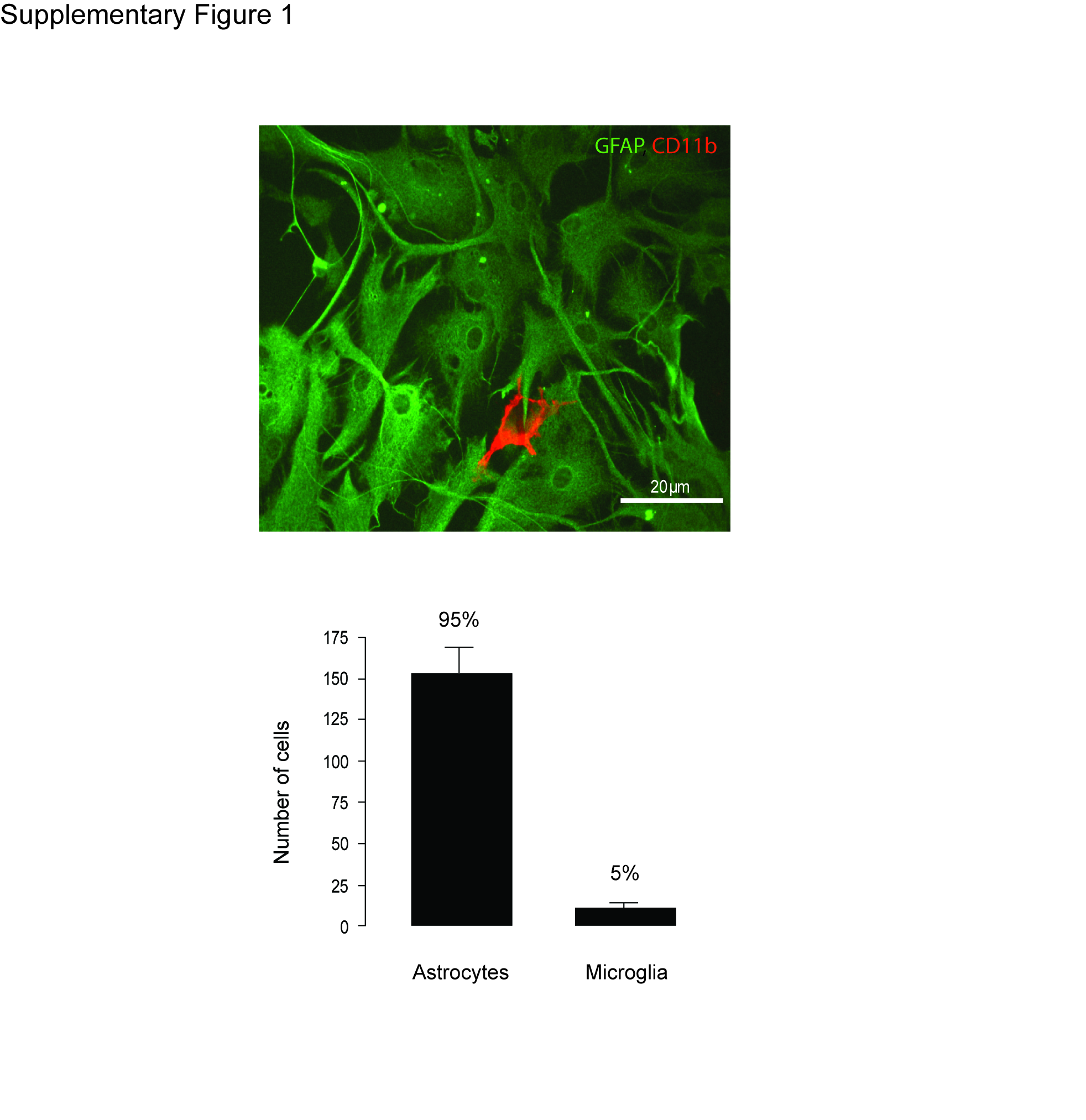

Supplement: Additional file 1: Figure S1. — Representative image (on top) showing the characteristic ratio of astrocytes-to-microglia in the cortical astrocytes culture. The cells were fixed in 4 % paraformaldehyde, immunostained for GFAP (astrocytes; in green) and CD11b (microglia; in red) and observed under a fluorescence microscope with a total magnification of ×1000. The graph below shows the average astrocyte cell number (~152) compared with the average microglial cell number (~10) after counting 10 microscopic fields in several 16 mm coverslips of astrocyte cultures in 4 independent experiments. The astrocyte purity was determined to be between 94 and 96 %. Scale bar corresponds to 20 μm. (TIF 16116 kb) [file 12974_2016_613_MOESM1_ESM.tif]

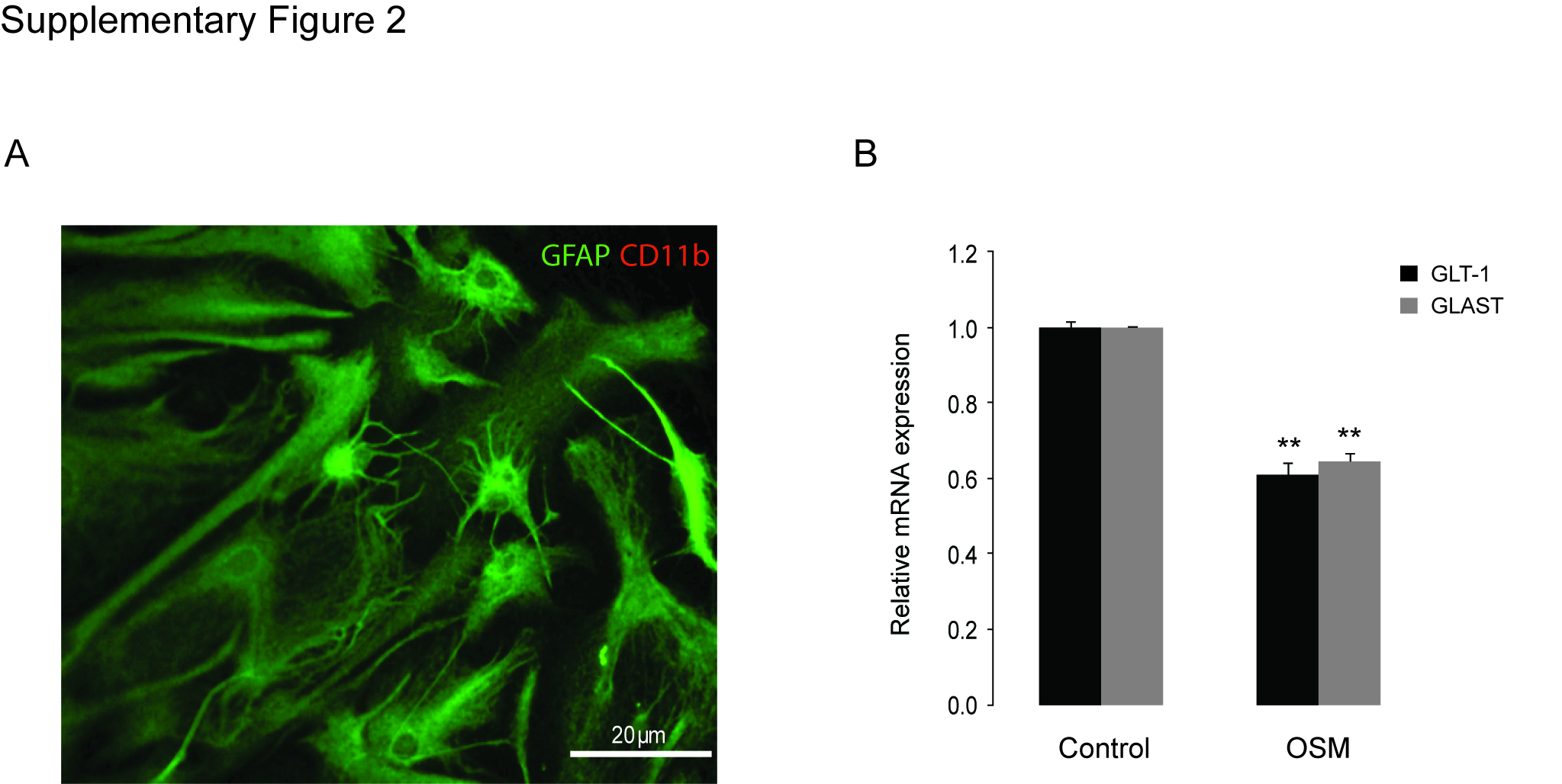

Supplement: Additional file 2: Figure S2. — OSM down-regulates GLT-1 and GLAST expression in microglia-depleted primary mouse cortical astrocytes. (A) Representative image showing the purity of cultured astrocytes following treatment with liposome clodronate (1 mg/mL for 4 h). The cells were washed with PBS, fixed in 4 % paraformaldehyde, immunostained for GFAP (astrocytes; in green) and CD11b (microglia; in red) and observed under a fluorescence microscope with a total magnification of ×1000. Scale bar corresponds to 20 μm. (B) Shows real-time PCR analyses of GLT-1 and GLAST mRNA (gene expression normalized to HPRT1) in control and OSM-treated (10 ng/mL for 24 h) astrocyte cultures. The cultures used in this experiment were treated with liposomal clodronate for 4 h, washed three times with PBS, and incubated in fresh culture media for 24 h, before addition of OSM. Data are normalized to untreated controls and presented as mean ± SEM; **p < 0.01, n = 3. (TIF 8798 kb) [file 12974_2016_613_MOESM2_ESM.tif]

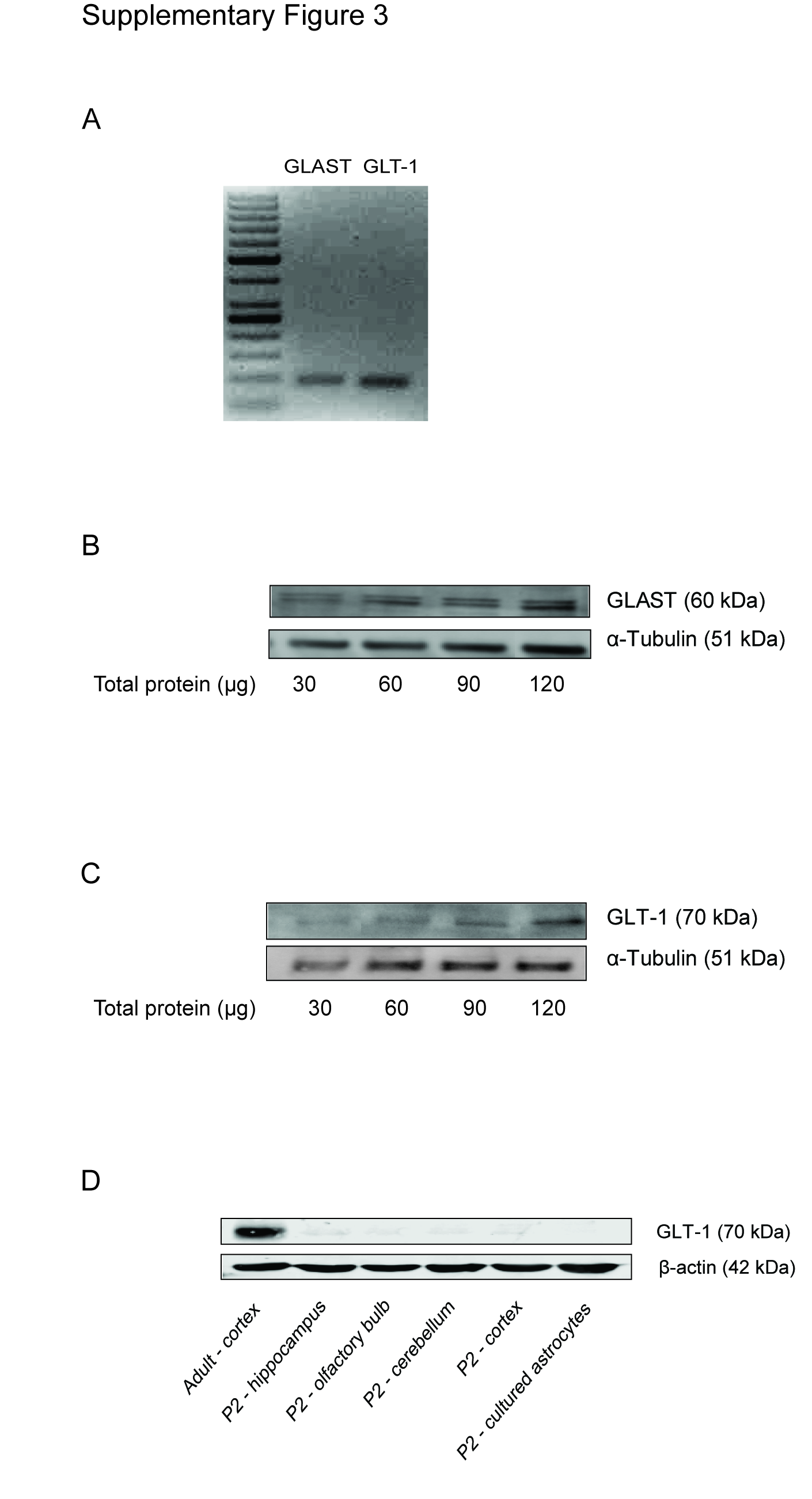

Supplement: Additional file 3: Figure S3. — Primary mouse astrocyte cultures differentially express GLAST and GLT-1 transporters. (A) Cortical astrocyte cultures were established from wild-type (C57BL/6J) mouse neonatal (P2) brains, and total RNA was isolated and analyzed for the expression of GLAST (91 bp) and GLT-1 (86 bp) mRNA by reverse transcriptase PCR (see Table 1 for primer details); GAPDH (not shown) served as the loading control. (B, C) Protein lysates prepared from cultured astrocytes were analyzed for GLAST (B) and GLT-1 (C) protein levels by Western blot. α-Tubulin served as the loading control. The observation that GLT-1 proteins were only faintly detected when high amounts of protein were loaded is consistent with the previously published findings that showed GLT-1 protein in astrocyte cultures is expressed at almost undetectable levels [55, 56]. (D) Shows Western blot analysis of GLT-1 proteins in lysates prepared from adult mouse cortex (12-week-old), neonatal (P2) brain regions (hippocampus, olfactory bulb, cerebellum and cortex), and cultured cortical astrocytes obtained from P2 mouse brains. As shown, GLT-1 proteins were abundantly expressed in the adult mouse cortex, whereas their expression level in different brain regions of P2 mouse brain was very low. β-Actin served as the loading control. (TIF 14907 kb) [file 12974_2016_613_MOESM3_ESM.tif]

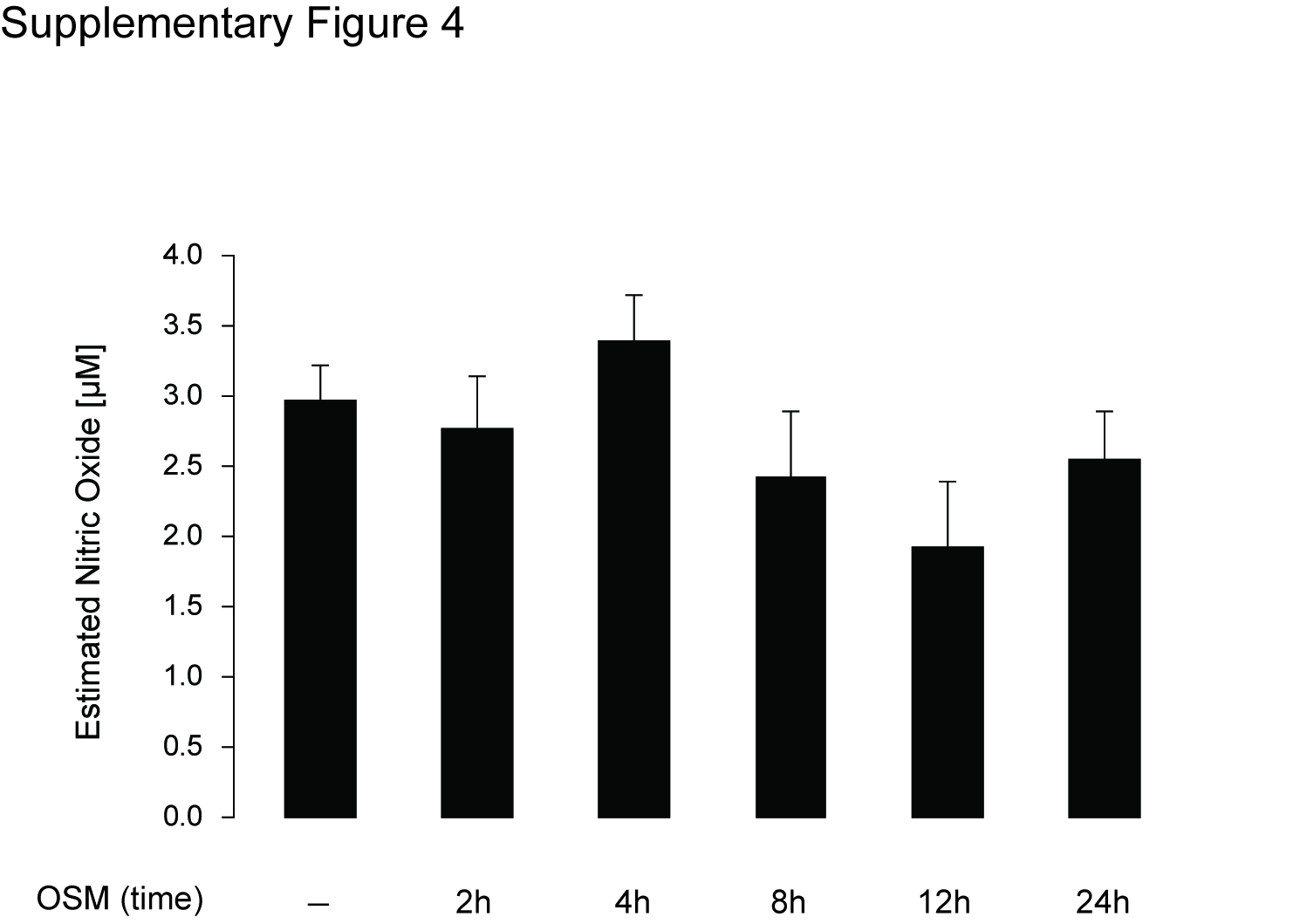

Supplement: Additional file 4: Figure S4. — OSM treatment does not induce nitric oxide (NO) release in cultured cortical astrocytes. Cortical astrocyte cultures from wild-type (C57BL/6J) mouse neonatal (P2) brains were treated without or with OSM (10 ng/mL) for 2, 4, 8, 12, and 24 h. Following OSM incubation, the supernatants were directly analyzed for NO content using Griess reagent system as per manufacturer’s protocol. Data represent absolute values of NO concentration and are mean ± SEM of two independent experiments performed in triplicates. (TIF 6742 kb) [file 12974_2016_613_MOESM4_ESM.tif]

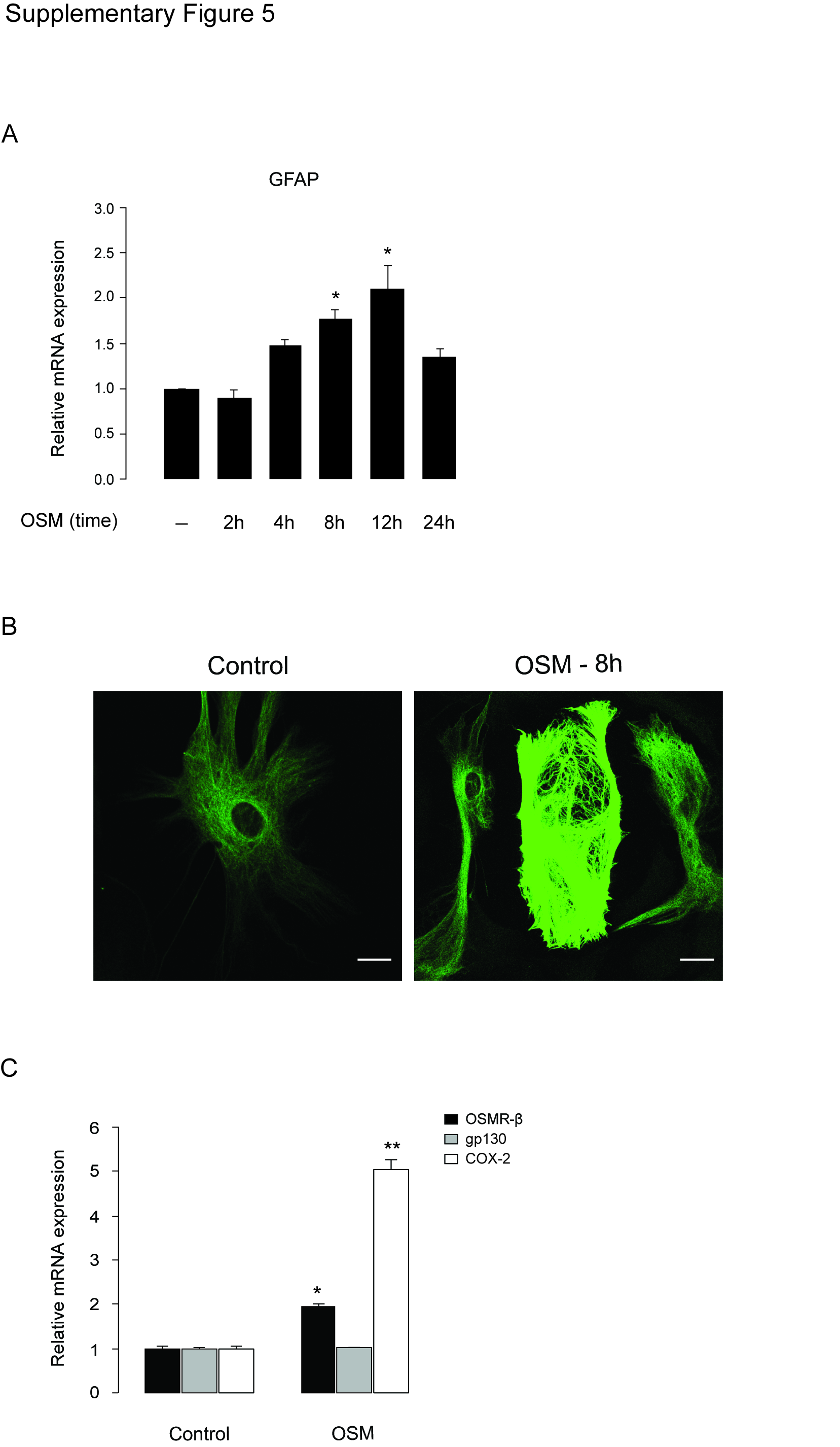

Supplement: Additional file 5: Figure S5. — OSM treatment induces GFAP, OSMR-β, and COX-2 gene expression in cultured cortical astrocytes. (A) Shows real-time PCR analysis of GFAP mRNA in primary astrocyte cultures that were treated without or with OSM (10 ng/mL) for 2, 4, 8, 12, and 24 h. GFAP gene expression was transiently induced by OSM, peaking at 8–12 h of treatment. *p < 0.05, n = 3. (B) Shows enhanced GFAP (green) immunoreactivity in cultured astrocytes following 8 h of OSM treatment (10 ng/mL), compared to the untreated control. Scale bar corresponds to 20 μm. (C) Shows real-time PCR analysis of OSMR-β, gp130, and COX-2 mRNA in primary astrocyte cultures that were treated without or with OSM (10 ng/mL) for 24 h. As shown, OSM induced two- and fivefold increase in OSMR-β and COX-2 mRNA, respectively. *p < 0.05, **p < 0.01, n = 3. (TIF 19046 kb) [file 12974_2016_613_MOESM5_ESM.tif]

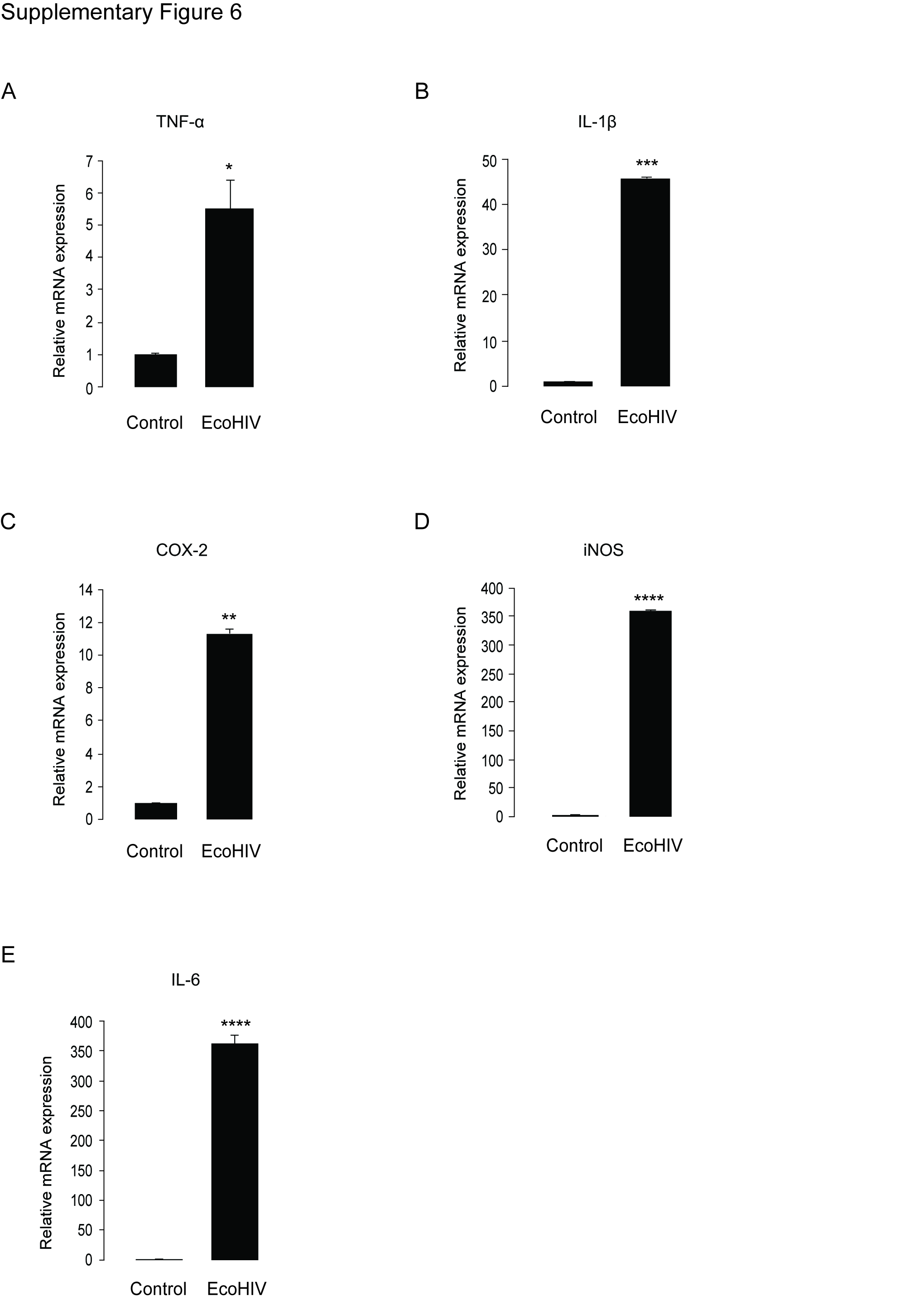

Supplement: Additional file 6: Figure S6. — EcoHIV infection induces the expression of pro-inflammatory genes in primary microglia. Real-time PCR analysis of TNF-α (A), IL-1β (B), COX-2 (C), iNOS (D), and IL-6 (E) mRNA were performed in cultured primary microglia that were infected without (control) or with EcoHIV (35,000 pg of p24, for 24 h). As shown, infection with EcoHIV induced approximately 5-, 45-, 11-, 350-, and 360-fold increase in TNF-α, IL-1β, COX-2, iNOS, and IL-6 mRNA, respectively. *p < 0.05, **p < 0.01, ***p < 0.001, ****p < 0.0001, n = 3. (TIF 23465 kb) [file 12974_2016_613_MOESM6_ESM.tif]

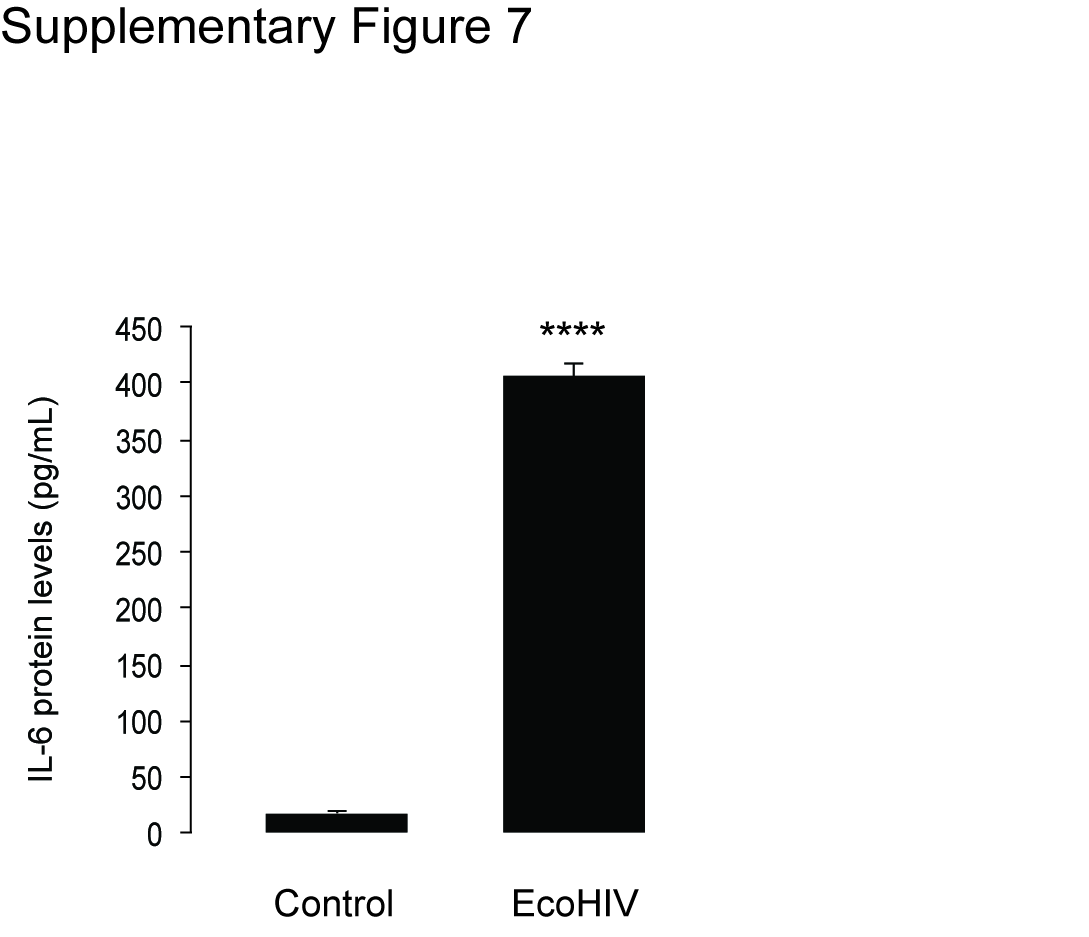

Supplement: Additional file 7: Figure S7. — EcoHIV induces IL-6 release in cultured primary mouse astrocytes. Secreted IL-6 proteins in culture supernatants from control and EcoHIV-infected (35,000 pg of p24, for 24 h) primary astrocytes were measured using a mouse IL-6 ELISA Ready-SET-Go kit (Affymetrix, eBioscience), following the manufacturer’s instructions. ****p < 0.0001, n = 6. (TIF 4562 kb) [file 12974_2016_613_MOESM7_ESM.tif]

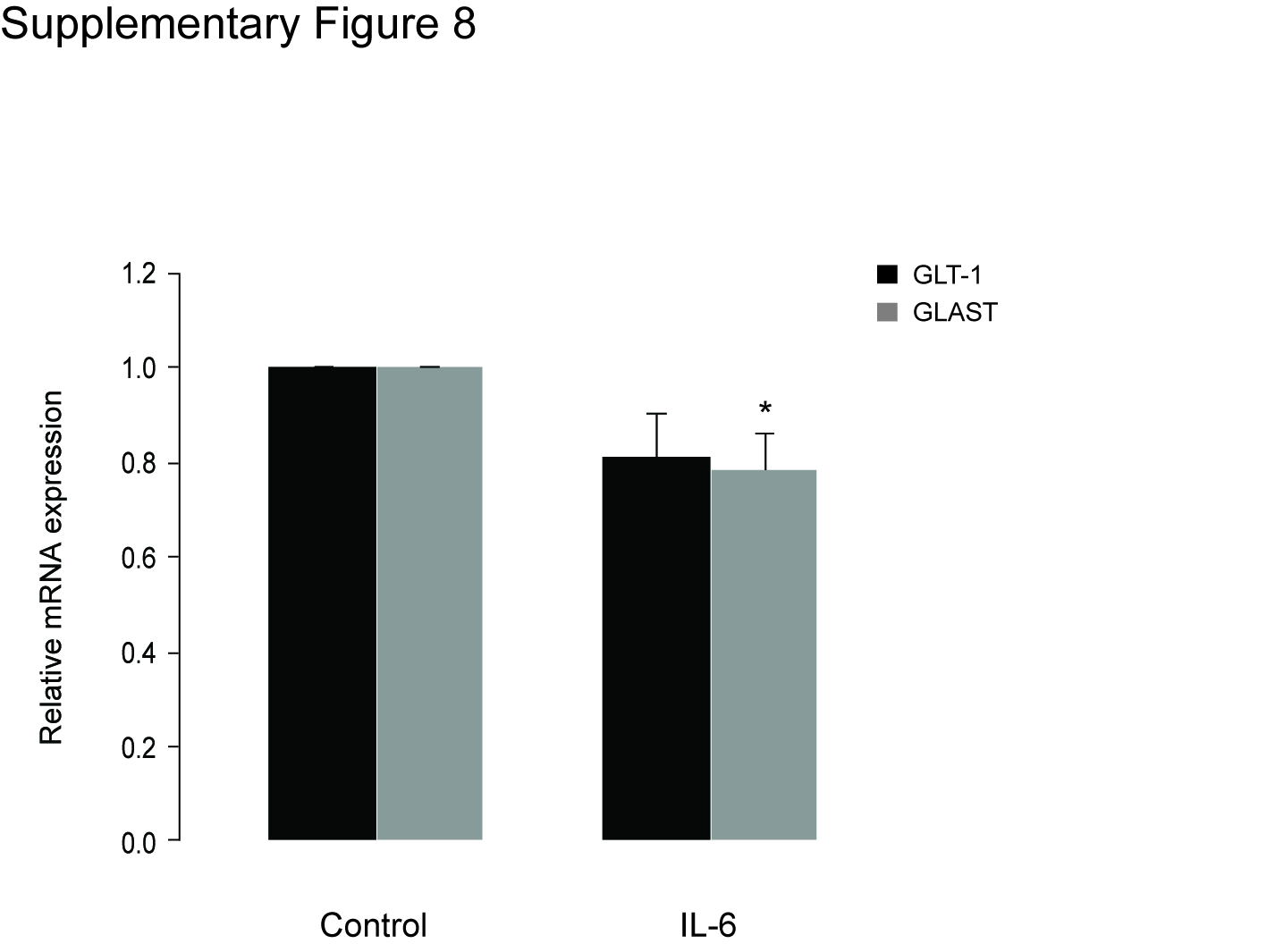

Supplement: Additional file 8: Figure S8. — IL-6 down-regulates GLAST, but not GLT-1, mRNA in primary mouse cortical astrocytes. Graph shows real-time PCR analyses of GLT-1 and GLAST mRNA (gene expression normalized to HPRT1) in control and IL-6-treated (10 ng/mL for 24 h) astrocyte cultures. Data are normalized to untreated controls and presented as mean ± SEM. *p = 0.04 (GLAST); p = 0.08 (GLT-1); n = 5; two-tailed Student’s t test. (TIF 6507 kb) [file 12974_2016_613_MOESM8_ESM.tif]
